# Arene Insertion Reactivity of α‑Diimine-Supported Cobalt(I) Hydrides

**DOI:** 10.1021/acs.organomet.5c00509

**Published:** 2026-04-06

**Authors:** Katherine L. Moffa, W. Neil Palmer, Matthew V. Pecoraro, Iraklis Pappas, Máté J. Bezdek, Paul J. Chirik

**Affiliations:** Department of Chemistry, 6740 Princeton University, Princeton, New Jersey 08544, United States

## Abstract

Exposure of cyclohexane solutions of (^iPr^DI)­Co­(η^3^-C_3_H_5_) (^iPr^DI = [2,6-^i^Pr_2_-C_6_H_3_N = C­(CH_3_)]_2_) to a H_2_ atmosphere in the presence of excess arene resulted in the formation of a transient cobalt hydride [(^iPr^DI)­Co–H], which formed the corresponding cobalt cyclohexadienyl compounds arising from arene insertion. Competing formation of a dinuclear bridging dicobalt dihydride, [(^iPr^DI)­Co­(H)]_2_, was identified and contributed to the observed modest yields. The dimeric structure of [(^iPr^DI)­Co­(H)]_2_ was confirmed in the solid state by X-ray diffraction, and the presence of two bridging hydride ligands was quantified by a Toepler pump experiment. The putative monomeric [(^iPr^DI)­Co–H] favored insertion at the *ortho* and *meta* positions of representative monosubstituted arenes. Reducing the size of the 2,6-aryl substituents on the α-diimine ligand from ^i^Pr to Me enabled the synthesis of [(^Mes^DI)­Co­(η^3^-C_3_H_5_)­(μ-N_2_)]_2_ (^Mes^DI = 2,4,6-Me_3_-C_6_H_3_N = C­(CH_3_)]_2_), which was characterized by X-ray diffraction. Exposure to H_2_ generated the putative [(^Mes^DI)­Co–H] whose reduced steric profile supported insertion of more sterically demanding arenes such as *tert*-butylbenzene and xylenes, with *ortho-* and *meta-*substituent site selectivity. Overall, the ligand donacity conferred by the α-diimine ligand promoted unique reactivity and selectivity on the process of arene insertion into a cobalt-hydride bond, as compared to reported bis­(phosphine) cobalt complexes.

## Graphical abstract

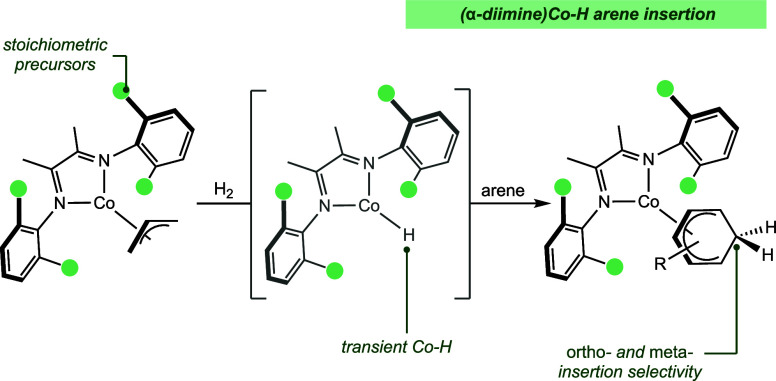

## Introduction

The insertion of an arene into a metal–hydride bond is a fundamental step in the hydrogenation of aromatic molecules mediated by transition metal catalysts.^1^ Although numerous examples of molecular and nanoparticulate metal compounds have been reported for catalytic arene hydrogenation, direct observation of arene insertion into a M–H bond is rare. Examples include complexes of iron,^2^ molybdenum,^3,4^ niobium,^5^ cobalt,^6,7^ and rhenium.^8^

Insight into the factors that determine arene insertion over competing η^6^-coodination, as well as those which determine site- and stereoselectivity of the insertion step are essential to the realization of methods for the selective reduction of arenes. To this end, our laboratory has reported arene insertion of electronically differentiated arenes into pincer-supported molybdenum dihydrides, where electron-rich arenes preferentially formed cyclohexadienyl complexes and electron deficient examples primarily formed the corresponding molybdenum arene complex.^4^

Jonas reported the generation of formally 14-electron cobalt­(I) hydrides supported by bis­(phosphine) ligands that promoted the insertion of benzene, toluene, and *p*-xylene to generate isolable η^5^-cyclohexadienyl compounds (Scheme 1A).^6^ With the prevalence of phosphine-modified cobalt compounds in catalytic alkene hydrogenation as well as their demonstrated use in catalytic arene hydrogenation,^9,10^ additional insight into the properties of the active cobalt­(I) hydride that support selective arene insertion were targeted. Our laboratory has investigated the kinetic and thermodynamic site selectivity of arene insertion into bis­(phosphine) cobalt hydrides using a host of CF_3_- and alkyl-substituted arenes. (*R,R*)-iPr-DuPhos was identified as an optimal supporting ligand, resulting in high thermodynamic selectivity for insertion at the *ipso*-CF_3_ position for mono-, 1,2-, and 1,3-disubstituted arenes. Protonation of the resulting diastereomeric η^5^-cyclohexadienyl compounds generated free cyclohexadiene products, which were isolated and used in Diels–Alder cycloaddition reactions (Scheme 1B).^7^

**Scheme 1:**
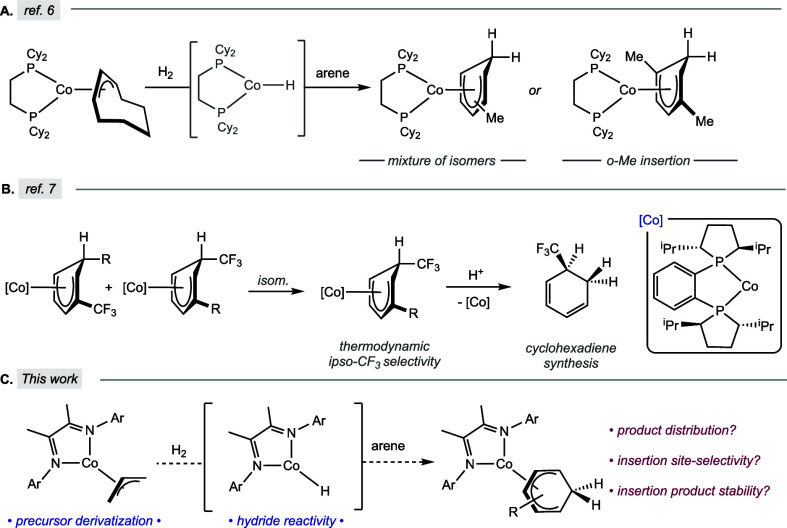
(A) Initial Report of Arene Insertion with In Situ-Generated Bis­(phosphine) Cobalt­(I) Hydrides,^6^ (B) Site-Selective Arene Insertion with In Situ-Generated Bis­(phosphine) Cobalt­(I) En Route to Cyclohexadienes,^7^ and (C) This Work: Arene Insertion with α-Diimine Cobalt Hydrides

The observation of site selective insertion with bis­(phosphine) cobalt complexes^7^ inspired study of other bidentate ligands with different electronic properties. Aryl-substituted α-diimine ligands were of interest given their low cost, straightforward and modular synthesis, and established redox-activity (Scheme 1C).^11^ The reported α-diimine cobalt­(I) allyl complex, (^iPr^DI)­Co­(η^3^-C_3_H_5_) has been used as an effective precatalyst for the catalytic hydroboration of tri-, tetra-, and geminally substituted alkenes using HBPin as the boron reagent.^12^ Here we describe a modified synthesis of (^iPr^DI)­Co­(η^3^-C_3_H_5_) and [(^Mes^DI)­Co­(η^3^-C_3_H_5_)­(μ-N_2_)]_2_ that upon treatment with H_2_ generated the transient and formally 14-electron cobalt hydrides that promoted arene insertion. The site selectivity of these reactions and product stability compared to the analogous bis­(phosphine) cobalt­(I) hydrides was established and a dimeric dicobalt hydride, [(^iPr^DI)­Co­(H)]_2_ was isolated and crystallographically characterized.

## Results and Discussion

### Synthesis and Hydrogenation of α-Diimine Cobalt Allyl Complexes

Using a modified literature procedure,^12^ (^iPr^DI)­Co­(η^3^-C_3_H_5_) was prepared by addition of (1,4-dioxane)­Mg­(C_3_H_5_)_2_ to a diethyl ether solution of (^iPr^DI)­CoCl_2_ at −35 °C. Warming to ambient temperature and stirring for 5 h followed by extraction with pentane provided (^iPr^DI)­Co­(η^3^-C_3_H_5_) as a teal powder in 86% yield (Scheme 2A)

**Scheme 2:**
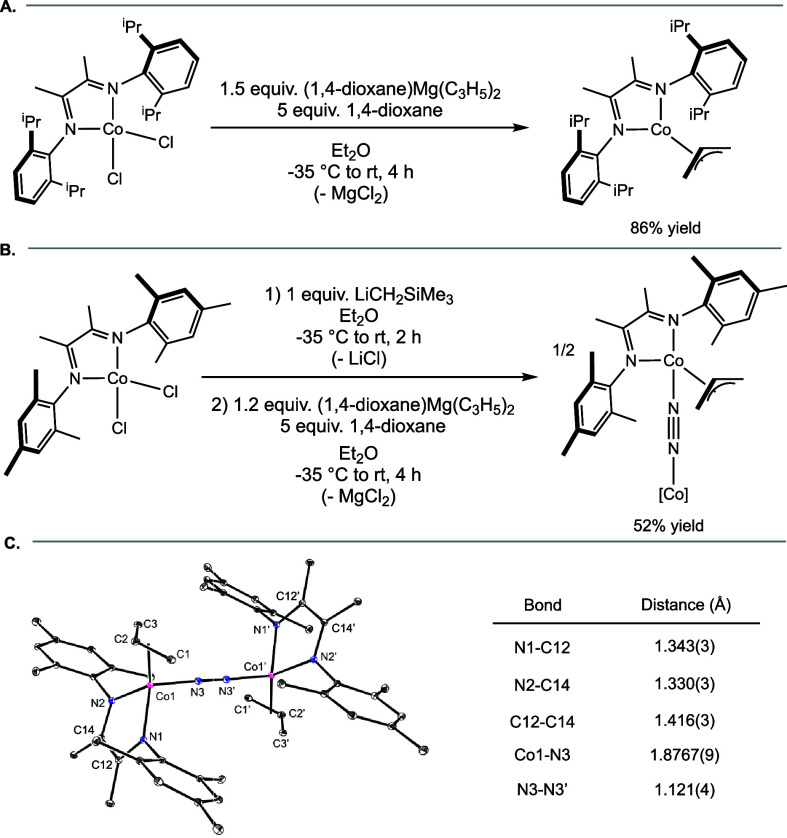
(A) Modified Synthesis of (^iPr^DI)­Co­(η^3^-C_3_H_5_); (B) Synthesis of [(^Mes^DI)­Co­(η^3^-C_3_H_5_)­(μ-N_2_)]_2_; (C) Representation of the Solid-State Structure of [(^Mes^DI)­Co­(η^3^-C_3_H_5_)­(μ-N_2_)]_2_ with Selected Bond Distances Reported in the Inset Table^a^

a 30% probability ellipsoids with hydrogen atoms omitted for clarity.

This procedure was applied to the synthesis of the analogous complex bearing *N*-mesityl substituents on the α-diimine, where treatment of (^Mes^DI)­CoCl_2_ with one equivalent of LiCH_2_SiMe_3_ prior to allylation with (1,4-dioxane)­Mg­(C_3_H_5_)_2_ proved optimal. This procedure furnished a deep orange solid identified as [(^Mes^DI)­Co­(η^3^-C_3_H_5_)­(μ-N_2_)]_2_ in 52% yield (Scheme 2B). Single crystals suitable for X-ray diffraction were obtained from a concentrated pentane solution stored at −35 °C. The solid-state structure is presented in Scheme 2C and established an overall *C*_2V_-symmetric dimeric structure containing a bridging, end-on N_2_ with two terminal η^3^ coordinated allyl ligands. The distortions to the bond lengths of the α-diimine ligand signaled redox-activity and one-electron reduction, where the C=N bond lengths were determined to be 1.343(3) and 1.330(3) Å, and the C–C bond length of the ligand backbone is 1.416(3) Å.^13^ These values are consistent with those previously reported for (^iPr^DI)­Co­(η^3^-C_3_H_5_), where C=N bond lengths were 1.329(3) Å and the C–C bond length was 1.427(4) Å.^12^ Thus, each monomer is best described as a low-spin cobalt­(II) ion engaged in antiferromagnetic coupling to a ligand radical anion.

Dissolving [(^Mes^DI)­Co­(η^3^-C_3_H_5_)­(μ-N_2_)]_2_ in cyclohexane-*d*_12_ generated a blue-purple solution that signaled dissociation of the N_2_ ligand.^14^ The resonance associated with the methyl groups of the imine backbone was assigned at −4.75 ppm, the upfield shift is consistent with participation of the ligand in the electronic structure of the compound. The allyl proton resonances were identified at −0.36 (*anti*), 10.14 (*syn*), and 10.99 ppm (*meso*). These assignments are comparable to those reported for (^iPr^DI)­Co­(η^3^-C_3_H_5_).^12^ An EXSY experiment at 23 °C established no crosspeaks between the *syn* and *anti* proton resonances, suggesting no η^3^,η^1^ interconversion of the allyl ligand on the NMR time scale.

Because multimetallic, polyhydrides were identified with bis­(phosphine) cobalt complexes and proved inactive for arene insertion chemistry,^6,7^ the hydrogenation of (^iPr^DI)­Co­(η^3^-C_3_H_5_) was conducted in the absence of arene. Exposure of a pentane solution of (^iPr^DI)­Co­(η^3^-C_3_H_5_) to 4 atm of H_2_ followed by removal of volatiles resulted in the isolation of a deep brown solid identified as [(^iPr^DI)­Co­(H)]_2_ (**[Co–H]**_**2**_) in 30% yield following recrystallization (Scheme 3A).

**Scheme 3:**
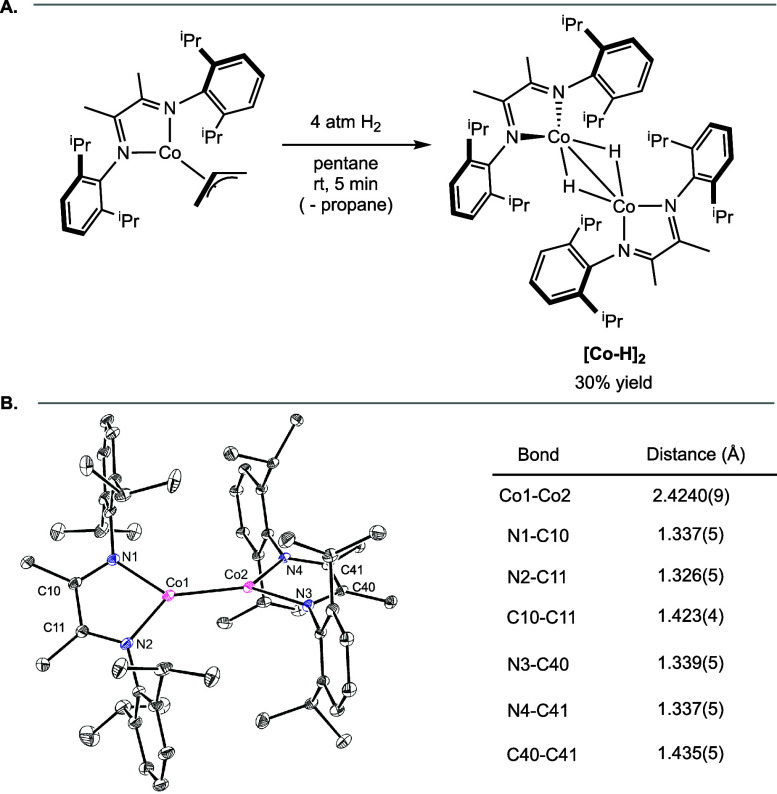
(A) Synthesis of [Co–H]_2_ and (B) Representation of the Solid-State Structure with Selected Bond Distances Reported in the Inset Table^a^

a 30% probability ellipsoid with hydrogen atoms omitted for clarity.

Broadened resonances between 4 and −71 ppm were observed in the cyclohexane-*d*_12_^1^H NMR spectrum of **[Co–H]**_**2**_, with the methyl groups on the imine backbone assigned to the resonance at −71 ppm. The solid-state structure was determined by X-ray diffraction and confirmed formation of a bimetallic cobalt compound, although the hydrides were not located. The [(^iPr^DI)­Co] planes were rotated relative to each other forming a dihedral angle of 43.12(14)°. The metrical parameters of the α-diimine ligands signaled redox-activity and that the supporting ligands were in their monoreduced state (Scheme 3B), supporting assignment of each cobalt center as cobalt­(II).^13^

Because the number of cobalt hydrides could not be reliably determined from X-ray diffraction or paramagnetic NMR spectroscopy, quantification of liberated H_2_ gas by Toepler pump was performed. A THF solution of **[Co–H]**_**2**_ was treated with excess (^iPr^DI)­CoCl_2_ to yield [(^iPr^DI)­CoCl]_2_ and free dihydrogen gas. The amount of H_2_ collected was consistent with two hydride ligands in total (one per cobalt) (Scheme S1). This result contrasts bis­(phosphine) bridging polyhydrides, where four to six hydride ligands were quantified based on Toepler pump experiments.^6,15^ This structure is also distinct from another α-diimine-supported cobaltate bridging polyhydride complex reported by Wolf, von Wangelin and co-workers, where three hydride ligands bridged two high spin cobalt­(II) centers.^16^ Benzene-*d*_6_ solutions of **[Co–H]**_**2**_ persisted overnight, indicating that this compound is inert to arene insertion and formation of a cobalt η^5^-cyclohexadienyl complex.

Arene coordination to the cobalt without insertion into the Co–H bond and subsequent H_2_ loss is also possible.^4^ An independent synthesis of (^iPr^DI)­Co­(η^6^-C_6_H_6_) was attempted following the literature procedure.^17^ Slow evaporation of a saturated solution produced single crystals identified by X-ray diffraction as a mixture of (^iPr^DI)­Co­(η^6^-C_6_H_6_) and [(^iPr^DI)­Co]_2_, where η^6^ coordination was observed to the imine aryl substituent of the other cobalt subunit. The benzene-*d*_6_^1^H NMR spectrum of this mixture exhibited several broad resonances between 15 and 0.5 ppm that were used to correlate with the general formation of formally cobalt(0) compounds, **Co­(0)** (Figure S5). Comparable crystallinities of these two complexes precluded isolation of each single component and identification of diagnostic spectroscopic features.

Similar hydrogenation experiments were conducted with [(^Mes^DI)­Co­(η^3^-C_3_H_5_)­(μ-N_2_)]_2_ in the absence of arene. Exposure of a cyclohexane-*d*_12_ solution of the compound to 4 atm of H_2_ resulted in complete conversion of the starting material over 5 min with formation of propane to yield a mixture of unidentified cobalt products (Figure S27). No evidence for a compound with ^1^H NMR spectroscopic features similar to **[Co–H]**_**2**_ was obtained.

### Arene Insertion Reactivity of (^iPr^DI)­Co­(η^3^-C_3_H_5_) and Characterization of Insertion Products

With spectroscopic signatures of potential byproducts established, the arene hydrodearomatization reactivity of (^iPr^DI)­Co­(η^3^-C_3_H_5_) was assayed (Scheme 4A). Treatment of a 4 mM pentane solution of (^iPr^DI)­Co­(η^3^-C_3_H_5_) containing 20 equiv of benzene with 1 atm of H_2_ at −196 °C resulted in a color change from teal to orange upon warming to ambient temperature and observation of ^1^H NMR signals corresponding to both **[Co–H]**_**2**_ (∼10%) and **Co­(0)**. Overlap of the resonances associated with **Co­(0)** with other signals precluded accurate quantification of this product. Resonances between 2 and 6 ppm as well as those between 0 and −0.5 ppm were also present and assigned to the cobalt cyclohexadienyl compound, (^iPr^DI)­Co­(η^5^-C_6_H_7_) (**Co1**), formed in 42% NMR yield and arising from benzene insertion into the putative hydride (Scheme 4B). The balance of cobalt is **Co­(0)** and unidentified precipitate.

**Scheme 4:**
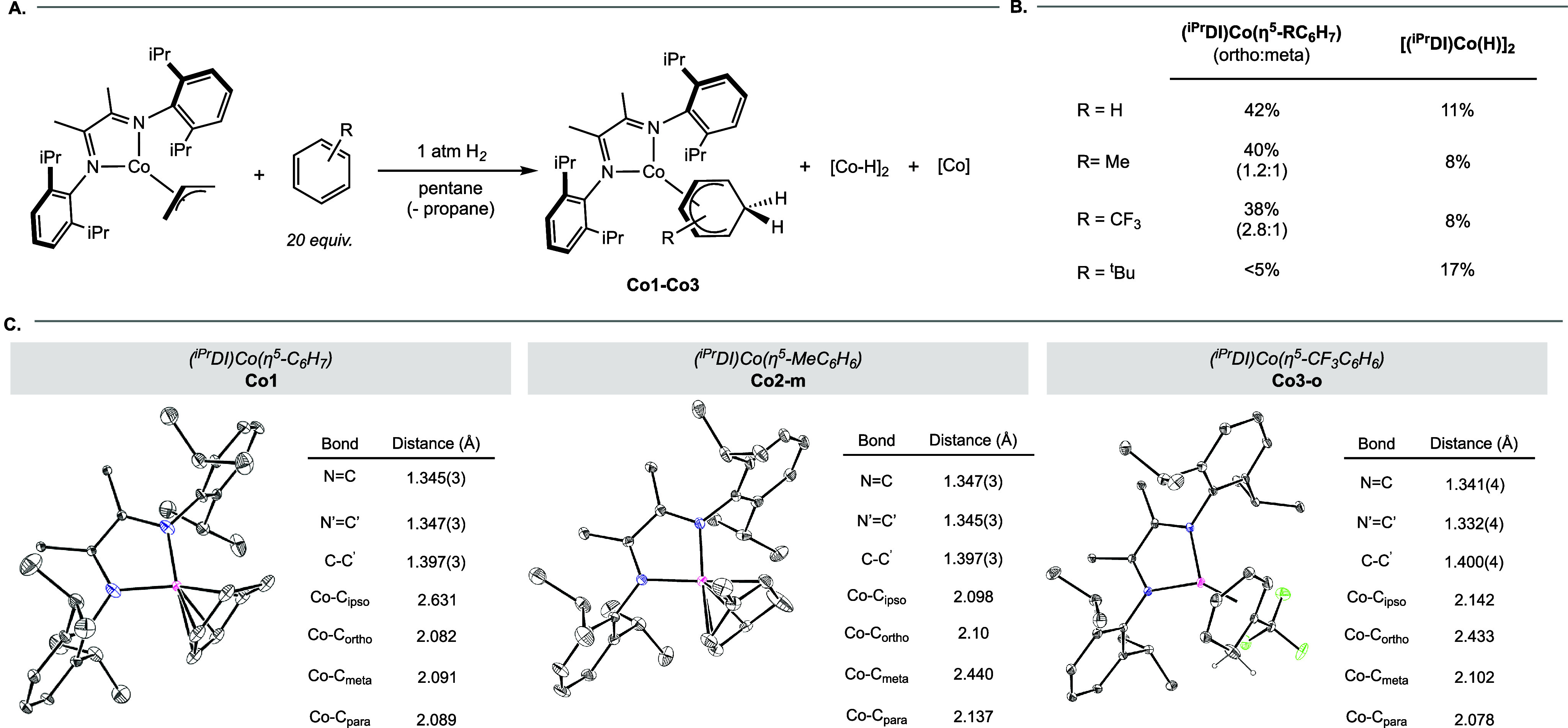
(A) Generation of ^iPr^DI-Supported Insertion Products and Anticipated Side Products, (B) Relative Yields of Insertion Compounds and Bridging Hydride with Representative Arenes^a^ and (C) Solid-state Structures of Selected Insertion Products with Selected Bond Distances Reported in the Inset Tables^b^ ^,^ ^c^

a Yields determined by ^1^H NMR spectroscopy using the 1,3,5-tris­(trifluoromethyl)­benzene standard.
b Representations of the solid-state structures with 30% probability ellipsoid with hydrogen atoms, except those on the *ipso* carbon of **Co3-o**, omitted for clarity.
c α-Diimine ligand distances were determined crystallographically, while Co-cyclohexadienyl ligand distances were determined using Mercury software.

The solid-state structure of **Co1** confirmed the presence of a cyclohexadienyl ligand. Single crystals suitable for X-ray diffraction were obtained by exposure of a neat benzene solution of (^iPr^DI)­Co­(η^3^-C_3_H_5_) to an atmosphere of H_2_, followed by removal of the volatiles and slow evaporation of pentane (Scheme 4C). Co–C­(sp^2^) distances were an average of 2.09 Å, while the Co–C­(sp^3^) distance corresponding to the site of insertion was 2.59(4) Å. The N1–C1 and N2–C2 bond lengths of the α-diimine chelate were 1.345(3) and 1.347(3) Å, respectively, while C1–C2 distance in the backbone was 1.397(3) Å. Taken together, these bond distances supported the ligand in its monoreduced form. The cyclohexane-*d*_12_^1^H NMR spectrum provided further insight into the electronic structure of this compound, as the resonance assigned to the imine methyl groups of **Co1** was identified at 0.52 ppm, shifted upfield and consistent with a ligand radical engaged in antiferromagnetic coupling to render the oxidation state assignment of the metal as cobalt­(II).

The insertion of monosubstituted arenes was then explored to examine the site selectivity of the hydrodearomatization reaction and the ratio of cobalt cyclohexadienyl product to **[Co–H]**_**2**_. When the same arene insertion procedure was conducted with toluene, approximately 40% of the cobalt cyclohexadienyl product was observed, arising from insertion at both the *ortho*- (**Co2-o**) and *meta*-positions (**Co2-m**) in a 1.2:1 relative ratio. These isomers were identified based on the number of cyclohexadienyl ligand resonances between 3 and 6 ppm in the ^1^H NMR spectrum. Approximately 8% of **[Co–H]**_**2**_ was observed along with **Co­(0)** products. Single crystals suitable for X-ray diffraction of **Co2-m** (Scheme 4C) were obtained in a similar manner to **Co1**. As with **Co1**, distorted α-diimine bond lengths consistent with a singly reduced chelate were observed, along with comparable Co–C distances to the cyclohexadienyl ligand. The methyl substituent was positioned perpendicular to the (^iPr^DI)Co plane, aligned with the center of the ligand.

Performing the arene insertion reaction with 20 equiv of α,α,α-trifluorotoluene yielded 38% of the *ortho-* and *meta-* insertion products (**Co3-o** and **Co3-m**) in a 2.8:1 ratio and 8% of **[Co–H]**_**2**_ observed by both ^1^H and ^19^F NMR spectroscopy. Single crystals of **Co3-o** were obtained by an identical procedure to that used with **Co1** and were characterized by X-ray diffraction (Scheme 4C). As with **Co1** and **Co2-m**, the α-diimine ligand of **Co3-o** exhibited α-diimine bond lengths consistent with a monoreduced chelate and comparable Co–C distances to the cyclohexadienyl ligand, whose CF_3_-substituent was also positioned perpendicular to the (^iPr^DI)Co plane as if to bisect the α-diimine chelate.

The exclusive formation of **Co3-o** and **Co3-m** highlighted that the arene insertion selectivity of the α-diimine cobalt complexes is under kinetic control. This selectivity is comparable to the initial isomer distribution observed with bis­(phosphine)-supported cobalt, where *ortho*- and *meta*-selectivity is observed prior to isomerization to the thermodynamically preferred *ipso*-CF_3_ position.^7^

The scope of the arene hydrodearomatization reaction was studied with arenes bearing larger alkyl substituents as well as disubstituted benzenes. With 20 equiv of *tert*-butyl benzene, <5% yield of the insertion product was obtained as judged by ^1^H NMR spectroscopy along with 17% of **[Co–H]**_**2**_, suggesting that the large aryl groups on the cobalt compound present a higher barrier for insertion of arenes bearing larger substituents. The insertions of *o*-, *m*-, and *p*-xylene were studied as representative disubstituted arenes and all produced **[Co–H]**_**2**_ as the only identifiable cobalt product. Additional substrates that did not result in the observation or formation of cobalt cyclohexadienyl products included: anisole, acetophenone, and methyl benzoate, where **[Co–H]**_**2**_ was the only observable reaction product or new product mixtures were observed as judged by ^1^H NMR spectroscopy.

The limited scope of arene insertion with transient [(^iPr^DI)­Co–H] prompted analogous studies with the less sterically encumbered cobalt precursor, [(^Mes^DI)­Co­(η^3^-C_3_H_5_)­(μ-N_2_)]_2_. Exposure of a cyclohexane-*d*_12_ solution of [(^Mes^DI)­Co­(η^3^-C_3_H_5_)­(μ-N_2_)]_2_ to 1 atm of H_2_ in the presence of 20 equiv of benzene per cobalt center resulted in conversion to the desired cobalt cyclohexadienyl compound, **Co4**, in 20% NMR yield along with unidentified cobalt products. This yield is reduced compared to the formation of (^iPr^DI)­Co­(η^5^-C_6_H_7_) (42%), demonstrating that the less sterically encumbered [(^Mes^DI)­Co–H] was subject to more rapid deleterious side reactivity (Scheme 5).

**Scheme 5:**
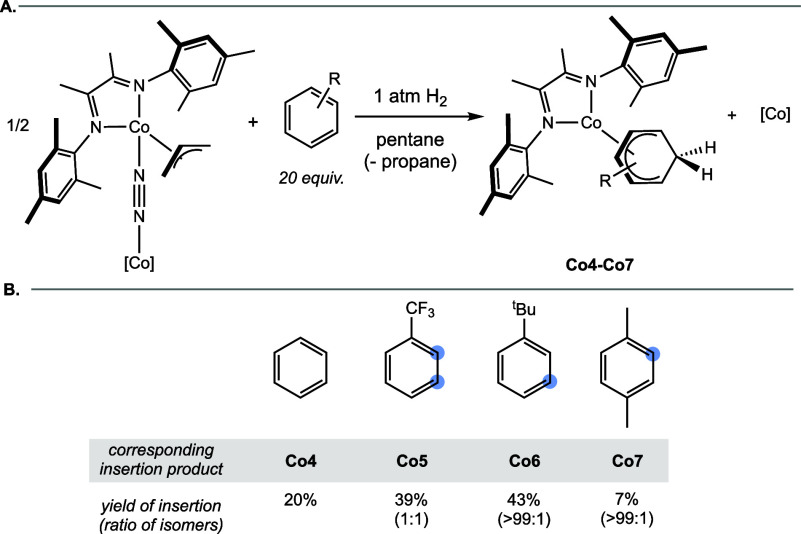
(A) Generation of ^Mes^DI-Supported Insertion Products and (B) Yields of Insertion Compounds with Representative Arenes^a^

a Yields determined by ^1^H NMR spectroscopy with a 1,3,5-tris­(trifluoromethyl)­benzene standard. Highlighted positions on the arene substrates indicate the observed site-selectivity of the hydrodearomatization reaction.

Attempts to characterize the products of this reaction by X-ray diffraction resulted in isolation of single crystals of the paramagnetic bis­(chelate) cobalt complex, (^Mes^DI)_2_Co. The structural characterization of this compound accounted for possible mass balance in this and other hydrogenation reactions.

Repeating the hydrodearomatization procedure with [(^Mes^DI)­Co­(η^3^-C_3_H_5_)­(μ-N_2_)]_2_ and 20 equiv of α,α,α-trifluorotoluene per cobalt center produced an equimolar amount of two cobalt cyclohexadienyl compounds as judged by ^1^H and ^19^F NMR spectroscopy. These were assigned as the *ortho*- (**Co5-o**) and *meta*- (**Co5-m**) insertion products, given the presence of desymmetrized resonances in the ^1^H NMR region diagnostic of hexadienyl protons. The 39% ^1^H NMR yield of the insertion compound was comparable to that observed following H_2_ activation of (^iPr^DI)­Co­(η^3^-C_3_H_5_), and enhanced *meta*-selectivity was observed (1:1 *ortho*:*meta* versus 2.8:1 *ortho*:*meta*). This indicated the steric environment afforded by the supporting ligand influences the site selectivity of the insertion reaction.

Insertion of *tert*-butylbenzene and a representative disubstituted benzene (*p*-xylene) was also studied as these reactions were unsuccessful with [(^iPr^DI)­Co–H]. Upon exposure of [(^Mes^DI)­Co­(η^3^-C_3_H_5_)­(μ-N_2_)]_2_ to the standard hydrogenation conditions in the presence of 20 equiv of *tert*-butylbenzene and *p*-xylene per cobalt center, 43 and 7% of the corresponding cobalt cyclohexadienyl products were observed by ^1^H NMR spectroscopy, respectively. The major isomer observed from the *tert*-butylbenzene insertion was assigned as the *meta*-insertion product by 2D ^1^H NMR spectroscopy. The major isomer of the cobalt cyclohexadienyl product observed from the *p*-xylene insertion product was assigned as the *ortho*-methyl isomer, based on the lack of symmetrical cyclohexadienyl resonances observed in the ^1^H NMR spectrum. The low insertion yield with *p*-xylene suggested that the rapid rate of side reactivity relative to productive arene insertion persisted even with the more open ^Mes^DI ligand. Concomitant formation of byproducts precluded isolation of these compounds and characterization beyond ^1^H NMR spectroscopy.

### Stability of Insertion Products

With kinetic insertion site selectivity assayed with both cobalt complexes, the isomerization behavior and thermodynamic selectivity of the insertion process was then evaluated. Over the course of 6 h at room temperature in cyclohexane-*d*_12_, the diagnostic peaks for **Co1** broadened and diminished with only resonances attributed to **[Co–H]**_**2**_ and **Co­(0)** observed after 72 h (Figure S28). Notably, the amount of **[Co–H]**_**2**_ remained constant over this time period. The observed product distribution suggests the cyclohexadienyl ligand is susceptible to β-hydride elimination to reform the free arene, followed by η^6^-coordination or dissociation to free arene is favored over-reinsertion and isomerization. Similar behavior was observed with a cyclohexane-*d*_12_ solution containing both **Co2-o** and **Co2-m** as the signals for these compounds diminished by 50% over the course of 72 h at room temperature. Again, the amount of **[Co–H]**_**2**_ remained constant over this time period while the signals for **Co­(0)** increased (Figure S29). Over 72 h at room temperature in cyclohexane-*d*_12_ solution, the amount of **Co3-o** and **[Co–H]**_**2**_ remained constant and **Co3-m** was depleted, evidenced by ^1^H NMR and ^19^F NMR spectroscopy (Figure S30). It was notable that **Co3-o** exhibited enhanced stability, demonstrating that cyclohexadienyl compounds derived from monosubstituted arenes with larger, electron-withdrawing substituents were more resistant to conversion to alternate cobalt products. Given the enhanced stability of **Co3-o**, the possibility of cyclohexadiene formation by protonation of the cyclohexadienyl ligand was evaluated. Treatment of a benzene-*d*_6_ solution of **Co3-o** with 2.5 equiv of benzoic acid resulted in an immediate color change from deep to light orange. Collection of the volatiles and analysis by ^19^F NMR spectroscopy identified formation of two new fluorine-containing organic products, one of which was the product formed upon protonation of the related ((*R,R*)-^i^Pr-DuPhos)­Co­(η^5^-CF_3_C_6_H_7_) compound (Figure S32).^7^ These organic fluorine-containing products were formed in <1% as judged by NMR spectroscopy.

The stability of the insertion products supported by the ^Mes^DI ligand was also evaluated. The benzene and *tert*-butyl benzene insertion compounds were almost completely consumed in cyclohexane-*d*_12_ solution over 24 h at room temperature. The cobalt compound derived from *p*-xylene insertion was completely depleted after 12 h at room temperature in cyclohexane-*d*_12_ solution. The α,α,α-trifluorotoluene example was stable in cyclohexane-*d*_12_ solution over the course of 24 h, with no identifiable loss in yield by ^1^H and ^19^F NMR spectroscopy.

Overall, the α-diimine-supported insertion products proved less stable than the corresponding bis­(phosphine)cobalt compounds, as reaction monitoring revealed consumption of insertion products rather than isomerization to thermodynamically preferred insertion sites, as in the case of the latter.^7^ This supported the necessity of a strong, electron-rich ligand field to stabilize hydride intermediates toward productive reactivity with arenes to favor reinsertion over conversion to alternative cobalt compounds.

## Conclusions

Treatment of the α-diimine-substituted cobalt allyl complexes, (^iPr^DI)­Co­(η^3^-C_3_H_5_) and [(^Mes^DI)­Co­(η^3^-C_3_H_5_)­(μ-N_2_)]_2_ with H_2_ in the presence of arenes resulted in the generation of transient [(α-diimine)­Co–H] compounds and arene insertion to form the corresponding cobalt η^5^-cyclohexadienyl derivatives. In the case of the more sterically protected cobalt complex, (^iPr^DI)­Co­(η^3^-C_3_H_5_), the cobalt hydride dimer [(^iPr^DI)­CoH]_2_ also formed, confirmed by hydrogenation reactions conducted in the absence of arene and by X-ray crystallography. The less substituted transient cobalt hydride, [(^Mes^DI)­Co–H] proved more prone to side reactivity as insertion yields were generally lower and an isolable cobalt hydride dimer was elusive. However, this complex did promote the insertion of more sterically hindered arenes such as *tert*-butylbenzene and *p*-xylene, substrates that were unreactive with in situ formed [(^iPr^DI)­Co–H]. Both transient hydrides favored insertion at the *ortho* and *meta* positions of the substituted arenes studied with enhanced solution-state stability observed for the α,α,α-trifluorotoluene derivatives. In summary, this study revealed arene insertion was accessible with bidentate ligands beyond bis­(phosphines) to yield products with differentiated site-selectivity as well as varied chemoselectivities and stabilities in solution.

## Supplementary Material



## Notes

Experimental data, including synthesis and characterization of cobalt complexes and NMR spectra (PDF)
