# Supplementary material for: Arene Insertion Reactivity of α‑Diimine-Supported Cobalt(I) Hydrides
Source: Organometallics. 2026 Apr 6;45(8):953–9. doi: 10.1021/acs.organomet.5c00509 (PMC13126669; doi:10.1021/acs.organomet.5c00509)
Supplement: Supplementary file 1 [file om5c00509_si_001.pdf]

Supporting Information

Arene Insertion Reactivity of α-Diimine-Supported Cobalt(I) Hydrides

*Katherine L. Moffa, W. Neil Palmer, Matthew V. Pecoraro, Iraklis Pappas, Máté J. Bezdek, and Paul J. Chirik**

Department of Chemistry, Frick Laboratory
Princeton University, Princeton, NJ 08544, USA

pchirik@princeton.edu

Table of Contents

General Considerations S2

Preparation of Cobalt Complexes S4

General Procedure and Products S7

Toepler Pump Procedure S12

Spectroscopic Data S13

Crystallographic Data S29

References S47

I. General Considerations

All air- and moisture-sensitive manipulations were carried out using vacuum line, Schlenk and cannula techniques or in an MBraun inert atmosphere nitrogen dry box unless otherwise noted. All glassware was stored in a pre-heated oven (≥ 150° C) prior to use. Pentane, benzene, toluene, diethyl ether, and tetrahydrofuran used for air- and moisture-sensitive manipulations were dried and deoxygenated using literature procedures.[1] Benzene-d_6 and cyclohexane-d_{12} used for NMR spectroscopy was distilled from sodium metal and stored over 4 Å molecular sieves. Celite and alumina were dried at 180 ºC under vacuum for 3 days prior to use in the glovebox.

1,4-dioxane was distilled from sodium and stored over 4 Å molecular sieves. Ethylbenzene, cumene, trifluorotoluene, and *tert*-butylbenzene were distilled from CaH_2 then filtered through an alumina plug. Biphenyl was dried on a vacuum line overnight. The following compounds and reagents were prepared according to literature procedures: ^{iPr}DI,[2] ^{Mes}DI,[2] (^{iPr}DI)$CoCl_2$,[3] [(^{Pr}DI)Co(Cl)]$_2$,[4] (^{Mes}DI)$CoCl_2$,[3] (^{iPr}DI)Co(η^3-C_3H_5),[5] (1,4-dioxane)Mg(allyl)$_2$.[6] H_2 gas was purchased from Airgas®, and passed through a column of alternating layers of 4 Å molecular sieves and MnO/Vermiculite before being introduced to glassware.

^{1}H NMR spectra were recorded on either Bruker AVANCE 300, 400 or 500 spectrophotometers operating at 300.13 MHz, 399.8 MHz and 500.46 MHz, respectively. ^{13}C NMR spectra were recorded on either Bruker Avance 300, 400 or 500 spectrometers operating at 75.48 MHz, 100.54 MHz and 125.85 MHz, respectively. All ^{1}H and ^{13}C NMR chemical shifts are reported in ppm relative to $SiMe_4$ using the ^{1}H and ^{13}C chemical shifts of the solvent as a standard.[7] ^{1}H NMR data for diamagnetic compounds are reported as follows: chemical shift, multiplicity (s = singlet, d = doublet, t = triplet, q = quartet, p = pentet, br = broad, m = multiplet, app = apparent, obsc = obscured), coupling constants (Hz), integration, assignment. ^{1}H NMR data for paramagnetic compounds are reported as follows: chemical shift, integration, peak width at half height (Hz). ^{13}C NMR data for diamagnetic compounds are reported as follows: chemical shift,

number of protons attached to carbon (e.g. CH_2), assignment. ^{19}F NMR spectra were recorded on Bruker Avance 400 or 500 spectrometers operating at 376.19 MHz and 470.96 MHz, respectively, and referenced to $CFCl_3$ as an external standard.

Single crystals suitable for X-ray diffraction were coated with polyisobutylene oil in the drybox, transferred to a nylon loop and then quickly transferred to either the goniometer head of a diffractometer equipped with a Bruker PHOTON III detector and Cu X-Ray tube (λ = 1.54178 Å) or a Rigaku XtaLAB Synergy-i equipped with a Mo X-ray tube (λ = 0.71073 Å) and a Cu X-ray tube (λ = 1.54178 Å). Preliminary data revealed the crystal system. The data collection strategy was optimized for completeness and redundancy using either the Bruker APEXII software suite or Rigaku CrysAlis^{Pro} software suite. The space group was identified, and the data were processed and corrected for absorption. The structures were solved using intrinsic phasing (SHELXT) and completed by subsequent Fourier synthesis and refined by full-matrix least-squares procedures in Olex2. Unless otherwise specified, hydrogen atoms were modelled as riding atoms.

II. Preparation of Cobalt Complexes.

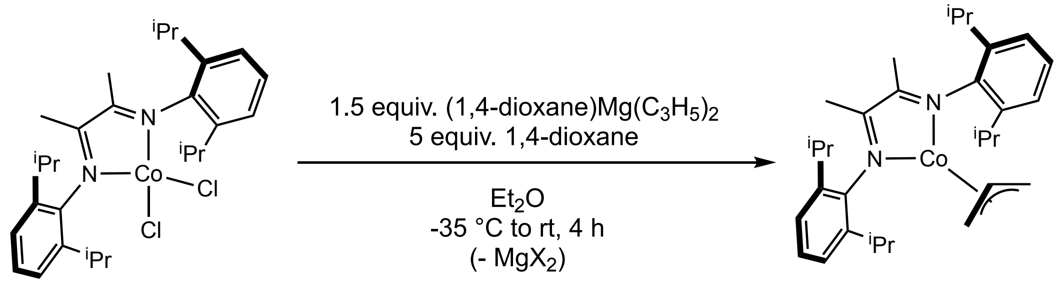

Preparation of (^{iPr}DI)Co(η^3-C_3H_5). To a 20 mL scintillation vial charged with a magnetic stirring bar and 0.15 g (^{iPr}DI)$CoCl_2$ (0.31 mmol) was added 10 mL Et_2O. The solution was cooled to -35°C. 0.066 g of (1,4-dioxane)Mg$(C_3H_5)_2$ (0.34 mmol, 1.1 equiv.) dissolved in 5 mL Et_2O containing 0.13 mL (5 equiv.) 1,4-dioxane was added dropwise while stirring and a color change from light to dark green was observed. The solution was stirred at room temperature for 5 hours. The volatiles were removed under vacuum and the resulting solid was taken up in 10 mL pentane and filtered through a pad of celite. Removal of solvent yielded 0.13 g (0.27 mmol, 86% yield) of a deep teal powder identified as (^{iPr}DI)Co(η^3-C_3H_5). Spectroscopic characterization was in agreement with reported values.[5]

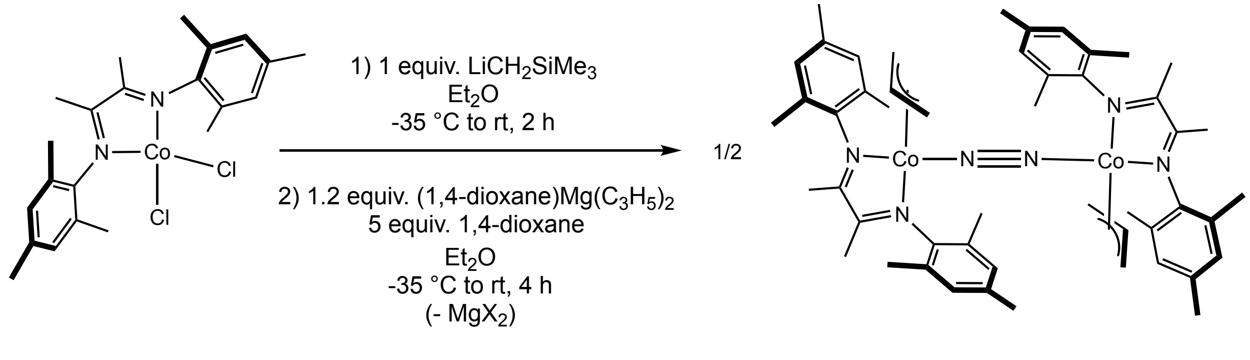

Preparation of [(^{Mes}DI)Co(η^3-C_3H_5)(μ-N_2)]$_2$. To a 20 mL scintillation vial charged with a magnetic stirring bar was added 0.20 g (0.44 mmol) of (^{Mes}DI)$CoCl_2$ and 10 mL of Et_2O. The resulting solution was cooled to -35 °C and a 5 mL Et_2O solution of 0.042 g $LiCH_2SiMe_3$ (0.44 mmol, 1 equiv.) was added dropwise with stirring. A color change from green to deep purple was observed. The solution was stirred at ambient temperature for 1 h. Solvent was removed in vacuo and the

resulting solid was dissolved in 10 mL of pentane and passed through a pipette celite plug. 0.17 g of a purple solid was recovered, considered for stoichiometry purposes to be $[(^{Mes}DI)Co(Cl)]_2$ (0.19 mmol). This solid was then dissolved in 10 mL of Et_2O. The solution was cooled to -35 °C, after which 0.044 g of (1,4-dioxane)$Mg(C_3H_5)_2$ (0.23 mmol, 1.2 equiv.) dissolved in 5 mL of Et_2O and 0.080 mL of 1,4-dioxane (0.94 mmol, 5 equiv.) was added dropwise while stirring. The solution remained dark but appeared to become more blue-hued. The solution was allowed to stir at room temperature for 5 hours. The volatiles were removed under vacuum, and the resulting solid was taken up in 5 mL pentane and filtered through a pad of celite. Concentration of the pentane to approximately 1 mL and storage at -35 °C overnight yielded 0.085 g (0.098 mmol, 52% yield) of a deep orange powder identified as $[(^{Mes}DI)Co(\eta^3\text{-}C_3H_5)(\mu\text{-}N_2)]_2$. Single crystals suitable for X-ray diffraction were obtained by slow evaporation of pentane at ambient temperature.

Note: This procedure proved to be incompatible with cobalt complexes bearing cyclohexyl and cyclopentyl-substituted alkyl α-diimine ligands, where the isolated products did not have ^{1}H NMR resonances consistent with those previously assigned to allylic protons. Moreover, no color change nor observable change in ^{1}H NMR resonances was observed upon exposure of these products to H_2. Attempts to crystallize these products instead resulted in isolation of bridging halide compounds.

^{1}H NMR (500 MHz, C_6D_{12}, 23 ºC): δ 10.99 (allyl H_{meso}, m, 1H), 10.14 (allyl H_{syn}, d, $^3J_{HH}$ = 5.0 Hz, 2H), 6.84 (aryl H, s, 4H), 2.51 (aryl *para*-CH_3, s, 6H), 1.63 (aryl *ortho*-CH_3, s, 12H), -0.36 (allyl H_{anti}, d, $^2J_{HH}$ = 20 Hz, 2H), -4.75 (imine CH_3, s, 6H). ^{13}C NMR (126 MHz, C_6D_{12}, 23 ºC): δ 166.09 (imine *C*=N), 163.65 (imine *C*-N), 133.37 (2,6-aryl *C*), 128.63 (aryl *C*H), 119.20 (4-aryl *C*), 33.54 (allyl *C*H_2), 28.4 (imine *C*H_3), 21.57 (aryl 4-*C*H_3), 18.73 (aryl 2,6-*C*H_3). Not located: allyl *C*H.

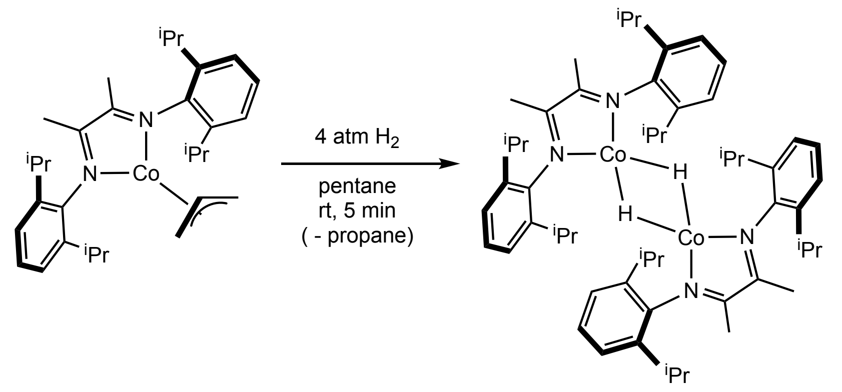

Preparation of $[Co\text{-}H]_2$. In a nitrogen filled glovebox, a 50 mL Schlenk flask was charged with a magnetic stirring bar. 0.069 g of $(^{iPr}DI)Co(\eta^3\text{-}C_3H_5)$ was dissolved in 2 mL pentane and added to the flask, which was then sealed and removed from the glovebox. 4 atm of H_2 were added and the mixture was allowed to thaw and stirred for 15 minutes at room temperature, and a color change from teal to black was observed. The reaction mixture was degassed with three freeze-pump-thaw cycles and returned to the glovebox, where the contents of the Schlenk flask were transferred to a 20 mL scintillation vial with additional pentane washes. The reaction mixture was concentrated to 2 mL of pentane and crystallized at -35 °C overnight, yielding 0.040 g (33% yield) of a brown-black crystalline solid identified as $[(^{iPr}DI)Co(H)]_2$. Single crystals suitable for X-ray diffraction were isolated by slow concentration in pentane.

^1H NMR (400 MHz, C_6D_{12}, 23 ºC): δ 4.87 (Δv = 17.45 Hz, 24 H, ^{i}Pr C*H*$_3$), -4.59 (Δv = 71.75 Hz, 8 H, ^{i}Pr C*H*), -5.18 (Δv = 20.50 Hz, 8H, aryl 2,6-C*H*), -8.23 (Δv = 8.25 Hz, 4H, aryl 4-C*H*), -31.21 (Δv = 39.66 Hz, 24 H, ^{i}Pr C*H*$_3$), -70.89 (Δv = 24.37 Hz, 12 H, imine C*H*$_3$). Not located: μ-H resonances.

III. General Procedures for Arene and Cobalt Cyclohexadienyl Products

General Procedure for Arene Insertion. In a typical experiment, in a nitrogen filled glovebox to 0.02 mmol of (DI)Co(η^3-C_3H_5) was added 20 equivalents of arene. The components were dissolved in 1 mL of pentane, yielding a ~42 mM solution of cobalt complex. The solution was transferred to a J. Young NMR tube and removed from the glovebox. The tube was submerged in liquid nitrogen and approximately 0.5 atm of H_2 was added. The solution was thawed and shaken to ensure adequate gas mixing. It was re-submerged in liquid nitrogen and the tube was degassed by with one freeze-pump-thaw cycle. The solvent was removed, and the reaction products were dissolved in 0.5 mL cyclohexane-d_{12} containing 1,3,5-tris-trifluoromethyl benzene as an internal standard. The resulting product was then analyzed by ^{1}H NMR and ^{19}F NMR spectroscopy if applicable. Note: parallel formation of paramagnetic products such as [(^{iPr}DI)Co(H)]$_2$ precluded isolation of analytically pure insertion products and multinuclear NMR spectroscopy was thus carried out on the product mixtures.

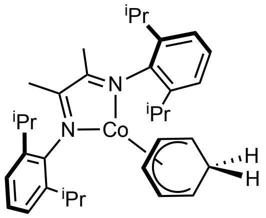

Co1. Prepared according to the general procedure using C_6H_6 and observed by ^{1}H NMR spectroscopy in 42% assay yield. Single crystals suitable for X-ray diffraction were isolated by slow concentration in pentane.

^{1}H NMR (300 MHz, C_6D_6, 23 ºC): δ 7.40 – 7.23 (m, 6H, aryl C*H*), 5.77 (t, $^3J_{HH}$ = 5.5 Hz, 1H, cyclohexadienyl C*H*), 4.62 (t, $^3J_{HH}$ = 6.1 Hz, 2H, cyclohexadienyl C*H*), 3.35 (h, $^3J_{HH}$ = 6.8 Hz, 4H, ^{i}Pr C*H*), 2.59 (t, $^3J_{HH}$ = 6.7 Hz, 2H, cyclohexadienyl C*H*), 2.46 (dt, $^3J_{HH}$ = 6.5 Hz, $^2J_{HH}$ = 6.9 Hz, 1H, cyclohexadienyl CH_2), 1.36 (d, $^3J_{HH}$ = 7.0 Hz, 12H, ^{i}Pr CH_3), 1.02 (d, $^3J_{HH}$ = 7.0 Hz, 12H, ^{i}Pr CH_3), 0.52 (s, 6H, imine CH_3), 0.18 (d, $^2J_{HH}$ = 14.2 Hz, 1H, cyclohexadienyl CH_2). ^{13}C NMR (126

MHz, C_6D_6, 23 °C): δ 154.7 (imine *C*=N), 144.8 (imine *C*-N), 139.8 (imine *C*-N), 125.8 (aryl *C*H), 123.5 (aryl *C*H), 92.2 (cyclohexadienyl *C*H), 79.7 (cyclohexadienyl *C*H), 41.5 (cyclohexadienyl *C*H), 27.9 (^{i}Pr *C*H), 26.0 (cyclohexadienyl *C*H_2), 24.9 (^{i}Pr *C*H_3), 24.2 (^{i}Pr *C*H_3), 19.6 (imine *C*H_3)

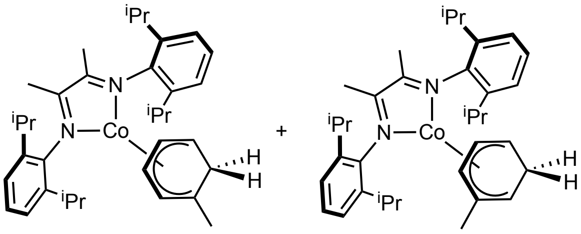

Co2. Prepared according to the general procedure using PhMe, consisting of two isomers (*ortho*- and *meta*- insertion), and observed by ^{1}H NMR spectroscopy in 40% assay yield. Single crystals suitable for X-ray diffraction were isolated by slow evaporation of pentane.

^{1}H NMR (400 MHz, C_6D_6, 23 °C): δ 7.39–7.19 (m, 12 H, overlapping aryl C*H*), 5.85 (d, $^{3}J_{HH}$ = 5.41 Hz, 1H, isomer a cyclohexadienyl C*H*), 5.54 (t, $^{3}J_{HH}$ = 5.39 Hz, 1H, isomer b cyclohexadienyl C*H*), 4.71 (t, $^{3}J_{HH}$ = 5.90 Hz, 1H, isomer b cyclohexadienyl C*H*), 4.39 (d, $^{3}J_{HH}$ = 5.46 Hz, 1H, isomer b cyclohexadienyl C*H*), 4.21 (t, $^{3}J_{HH}$ = 6.44 Hz, isomer a cyclohexadienyl C*H*), 4.15 (h, $^{3}J_{HH}$ = 6.85 Hz, 2H, ^{i}Pr C*H*), 3.93 (h, $^{3}J_{HH}$ = 6.89 Hz, 2H, ^{i}Pr C*H*), 2.86 (t, $^{3}J_{HH}$ = 5.46 Hz, 1H, isomer a cyclohexadienyl C*H*), 2.82-2.58 (m, 4H, overlapping ^{i}Pr C*H*), 2.49 (m, overlapping isomer a cyclohexadienyl C*H* and C*H_2*), 2.40 (m, isomer b cyclohexadienyl C*H_2*), 2.21 (t, $^{3}J_{HH}$ = 5.46 Hz, isomer b cyclohexadienyl C*H*), 1.60 (m, 12H overlapping ^{i}Pr C*H_3*), 1.26 (m, 12H, overlapping ^{i}Pr C*H_3*), 0.49 (s, 6H, imine C*H_3*), 0.43 (s, 6H, imine C*H_3*), 0.30 (d, $^{2}J_{HH}$ = 16.61 Hz, 1H, isomer a cyclohexadienyl C*H_2*), 0.24 (d, $^{2}J_{HH}$ = 13.21 Hz, 1H, isomer b cyclohexadienyl C*H_2*). Not located: 2x inserted arene CH_3.

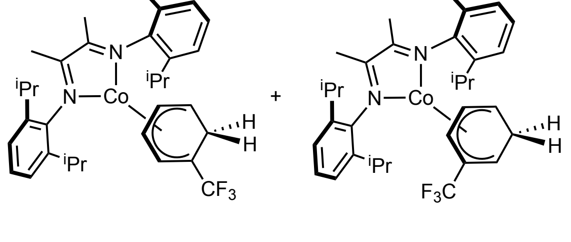

Co3. Prepared according to the general procedure using α,α,α-trifluorotoluene consisting of two isomers (*ortho*- and *meta*- insertion) and observed by ^{1}H NMR in 38% NMR yield. Single crystals of the *ortho*-isomer suitable for X-ray diffraction were isolated by slow concentration in pentane.

^{1}H NMR (400 MHz, cyclohexane-d_{12}, 23 °C): δ 7.30–6.93 (m, 12H, overlapping isomer a and b aryl C*H*), 6.02 (d, $^3J_{HH}$ = 6.0 Hz, 1H, m isomer cyclohexadineyl C*H*), 5.33 (t, $^3J_{HH}$ = 6.0 Hz, 1H, o isomer cyclohexadienyl C*H*), 5.00 (d, $^3J_{HH}$ = 7.2 Hz, 1H, o isomer cyclohexadienyl C*H*), 4.92 (t, $^3J_{HH}$ = 6.7 Hz, 1H, o isomer cyclohexadienyl C*H*), 3.95 (m, 2H, ^{i}Pr C*H*), 3.76 (m, 2H, ^{i}Pr C*H*), 2.98 (t, $^3J_{HH}$ = 7.2 Hz, 1H, o isomer cyclohexadienyl C*H*), 2.71 (m, 2H, ^{i}Pr C*H*), 2.62-2.50 (m, overlapping ^{i}Pr C*H* and isomer b cyclohexadienyl C*H*), 2.38-2.27 (m, 1H, m isomer cyclohexadienyl C*H*$_2$), 2.10 (m, 1H, m isomer cyclohexadienyl C*H*), 2.13 (t, $^3J_{HH}$ = 7.2 Hz, 1H, o isomer cyclohexadienyl C*H*), 1.52 (d, $^3J_{HH}$ = 7.3 Hz, 6H, iPr C*H*$_3$), 1.20 (d, $^3J_{HH}$ = 6.7 Hz, 6H, iPr C*H*$_3$), 1.05 (d, $^3J_{HH}$ = 6.8 Hz, 6H, iPr C*H*$_3$), 0.95 (d, $^3J_{HH}$ = 6.9 Hz, 6H, iPr C*H*$_3$), 0.69 (s, 3H, m isomer imine C*H*$_3$), 0.63 (s, 3H, o isomer imine C*H*$_3$), 0.28 (d, $^2J_{HH}$ = 13.1 Hz, 1H, o isomer cyclohexadienyl C*H*$_2$), 0.04 (d, $^3J_{HH}$ = 14.3 Hz, 1H, m isomer cyclohexadienyl C*H*$_2$).

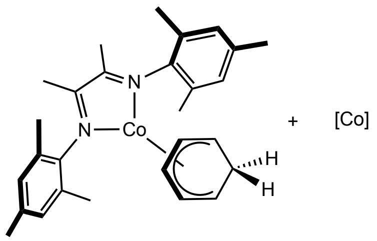

Co4. Prepared according to the general procedure using C_6H_6 and observed by ^{1}H NMR spectroscopy in 20% assay yield.

^{1}H NMR (400 MHz, C_6D_{12}, 23 °C): δ 6.93 (s, 4H, aryl C*H*), 5.44 (t, $^3J_{HH}$ = 4.75 Hz, 1H, cyclohexadienyl C*H*), 4.28 (t, $^3J_{HH}$ = 6.39 Hz, 2H, cyclohexadienyl C*H*), 2.91 (t, $^3J_{HH}$ = 6.39 Hz, 2H, cyclohexadienyl C*H*), 2.36 (s, 6H, 4-aryl C*H*$_3$), 2.23 (m, 2H, cyclohexadienyl C*H*), 2.09 (s, 12H, 2,6-aryl C*H*$_3$), 0.55 (s, 6H, imine C*H*$_3$), 0.08 (d, $^2J_{HH}$ = 13.9 Hz, 1H, cyclohexadienyl C*H*$_2$).
^{13}CNMR (126 MHz, C_6D_{12}, 23 °C): 155.5 (imine *C*=N), 143.5 (imine *C*-N), 133.40 (aryl *C*), 129.0

(aryl *C*H), 128.5 (aryl *C*), 93.0 (cyclohexadienyl *C*H), 80.8 (cyclohexadienyl *C*H), 41.6 (cyclohexadienyl *C*H), 21.4 (aryl CH_3), 18.4 (aryl CH_3), 17.3 (imine CH_3). Not located: cyclohexadienyl CH_2.

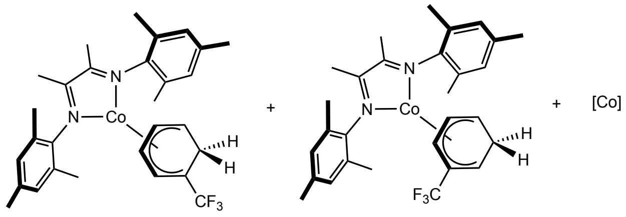

Co5. Prepared according to the general procedure using α,α,α-trifluorotoluene and observed by ^{1}H NMR in 39% assay yield. Isomers were assigned relative to each other in the ^{1}H NMR spectrum. ^{1}H NMR (400 MHz, cyclohexane-d_{12}, 23 °C): δ 7.00 (s, 4H, isomer a aryl C*H*), 6.87 (s, 4H, isomer b aryl C*H*), 5.73 (d, $^3J_{HH}$ = 5.58 Hz, 1H, isomer b hexadienyl C*H*), 5.30 (t, $^3J_{HH}$ = 4.8 Hz, 1H, isomer a cyclohexadienyl C*H*), 4.85 (t, $^3J_{HH}$ = 6.4 Hz, 1H, isomer b cyclohexadienyl C*H*), 4.66 (t, $^3J_{HH}$ = 5.4 Hz, 1H, isomer a cyclohexadienyl C*H*), 4.01 (t, $^3J_{HH}$ = 4.0 Hz, 1H, isomer b cyclohexadienyl C*H*), 2.91 (t, $^3J_{HH}$ = 4.8 Hz, 1H, isomer a cyclohexadienyl C*H*), 2.35 (s, 12H, aryl CH_3), 2.30 (s, 12H, aryl CH_3), 2.26-2.22 (m, 2H, 2 overlapping isomer a and b cyclohexadienyl CH_2), 1.85 (s, 12H, 2 overlapping aryl CH_3), 0.63 (s, 6H, imine CH_3), 0.55 (s, 6H, imine CH_3), 0.25 (d, $^2J_{HH}$ = 13.9 Hz, 1H, isomer b cyclohexadienyl CH_2), 0.18 (d, $^2J_{HH}$ = 14.3 Hz, 1H, isomer a cyclohexadienyl CH_2).

^{19}F NMR (376 MHz, cyclohexane-d_{12}, 23 °C): δ. 60.78 (s, 3F isomer a), 62.46 (s, 3F, isomer b). Note: these are relative assignments and do not necessarily correspond to isomer a and b identified by ^{1}H NMR.

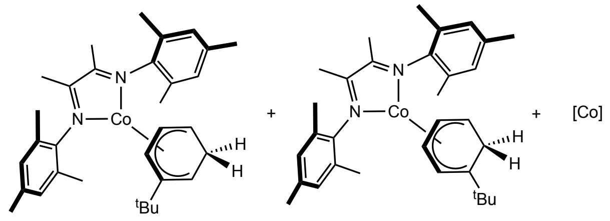

major

Co6. Prepared according to the general procedure using *tert*-butylbenzene and observed by ^{1}H NMR in 43% assay yield.

^{1}H NMR (400 MHz, cyclohexane-d_{12}, 23 ºC): δ 7.01 (s, 2H, aryl C*H*), 6.91 (s, 2H, aryl C*H*), 5.82 (d, $^{3}J_{HH}$ = 5.57 Hz, 1H, cyclohexadienyl C*H*), 3.96 (t, $^{3}J_{HH}$ = 6.34 Hz, 1H, cyclohexadienyl CH), 2.79 (t, $^{3}J_{HH}$ = 6.68 Hz, 1H, cyclohexadienyl CH), 2.40 (s, 9H, cyclohexadienyl ^{t}Bu CH_3), 2.26 (dt, $^{2}J_{HH}$ = 13.41 Hz, $^{3}J_{HH}$ = 6.28 Hz, 1H, cyclohexadienyl CH_2), 1.92 (m, 4H, ^{i}Pr C*H*) 1.34 (s, 12H, ^{i}Pr CH_3), 1.08 (s, 12H, ^{i}Pr CH_3), 0.49 (s, 6H, imine CH_3), -0.03 (d, $^{2}J_{HH}$ = 14.15 Hz, 1H, cyclohexadienyl CH_2)

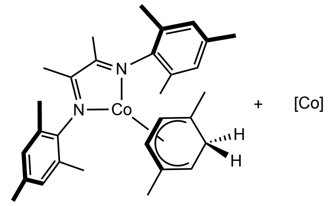

Co7. Prepared according to the general procedure using *p*-xylene and observed by ^{1}H NMR in approximately 7% assay yield. After recognition of diagnostic cyclohexadienyl resonances in the ^{1}H and ^{13}C NMR, further characterization was not pursued due to low quantities of the proposed insertion product.

IV. Toepler Pump Experiments

To a 100 mL Schlenk flask charged with a magnetic stir bar was one GC vial containing 0.092 g (0.0988 mmol) of **[(^{iPr}DI)Co(H)]$_2$** and 0.114g (0.213 mmol, 2.1 equiv.) of **(^{iPr}DI)CoCl$_2$.** 15 mL of anhydrous THF was thoroughly degassed using three freeze-pump-thaw cycles to ensure no extraneous gas was introduced into the system. The THF was distilled into the flask containing the GC vials and the vessel was sealed. These were thawed and the resulting slurry stirred for 10 minutes, where a rapid color change from brown to green was observed and along with vigorous bubbling. The contents of the vessel were again frozen and the generated gas was exposed to the Toepler pump and collected over approximately 30 cycles (15 minutes). The freeze-pump-thaw procedure ofthe reaction vessel was repeated until mercury levels after each 30-cycle period returned a consistent reading (10 times). A mercury level difference of 92 mm was observed, corresponding to 0.0995 mmol H_2 and supporting one hydride ligand per cobalt center.

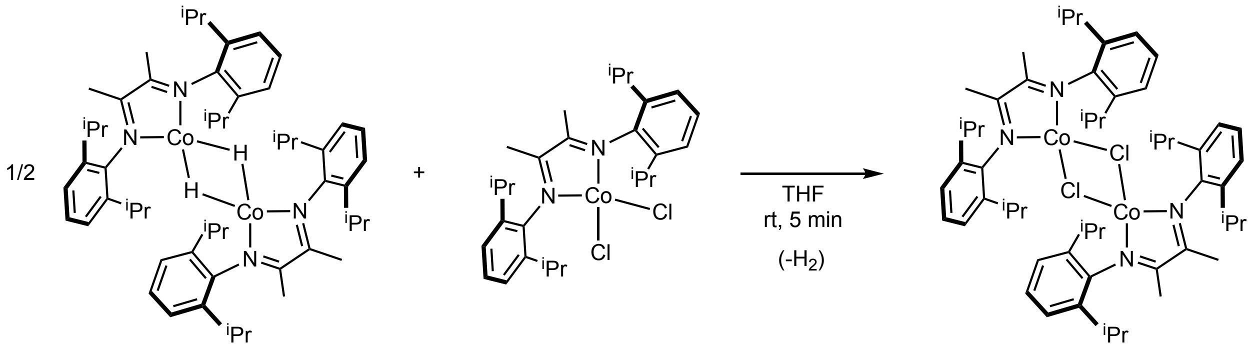

Scheme S1. Comproportionation reaction used for hydride quantification by Toepler pump.

V. Spectroscopic Data

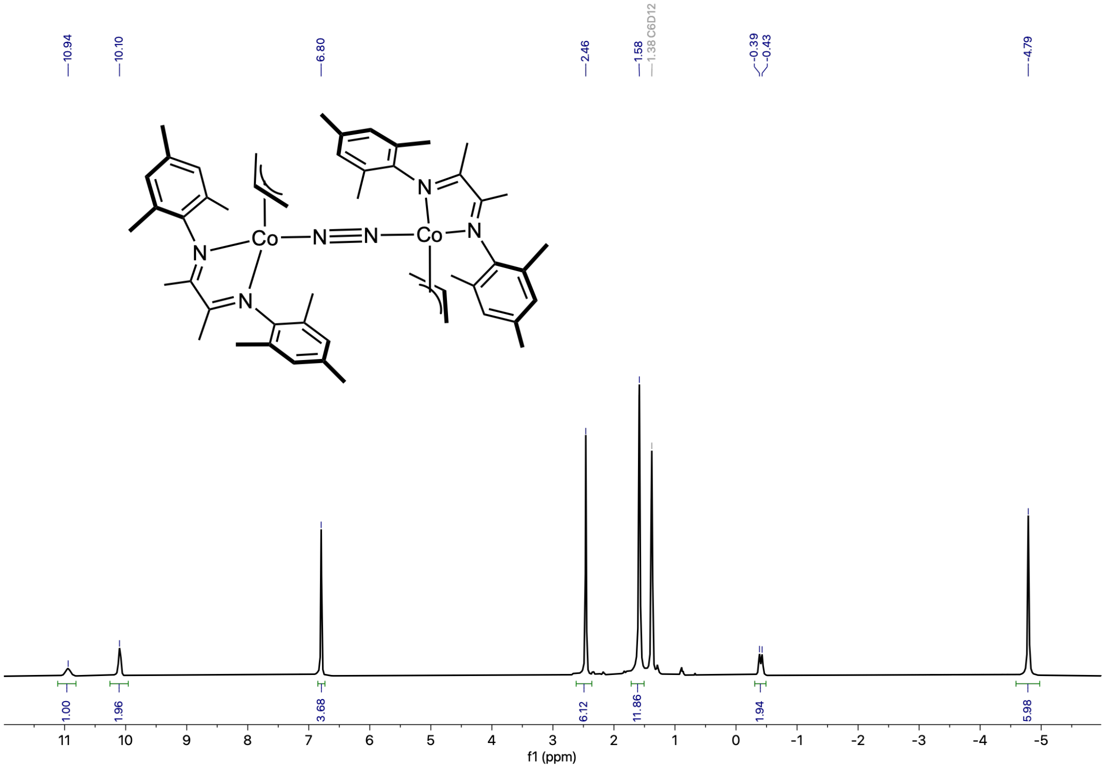

Figure S1. Representative ^{1}H NMR spectrum of (^{Mes}DI)Co(η^3-C_3H_5) in cyclohexane-d_{12}.

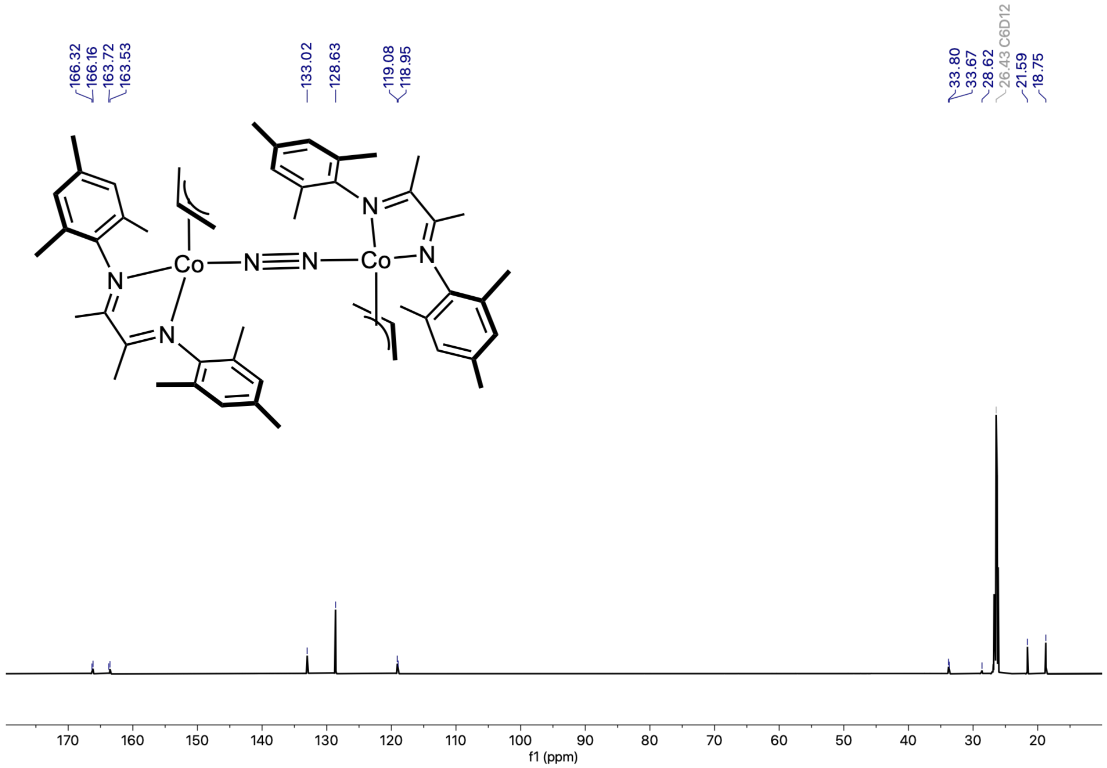

Figure S2. Representative ^{13}C NMR spectrum of (^{Mes}DI)Co(η^3-C_3H_5) in cyclohexane-d_{12}.

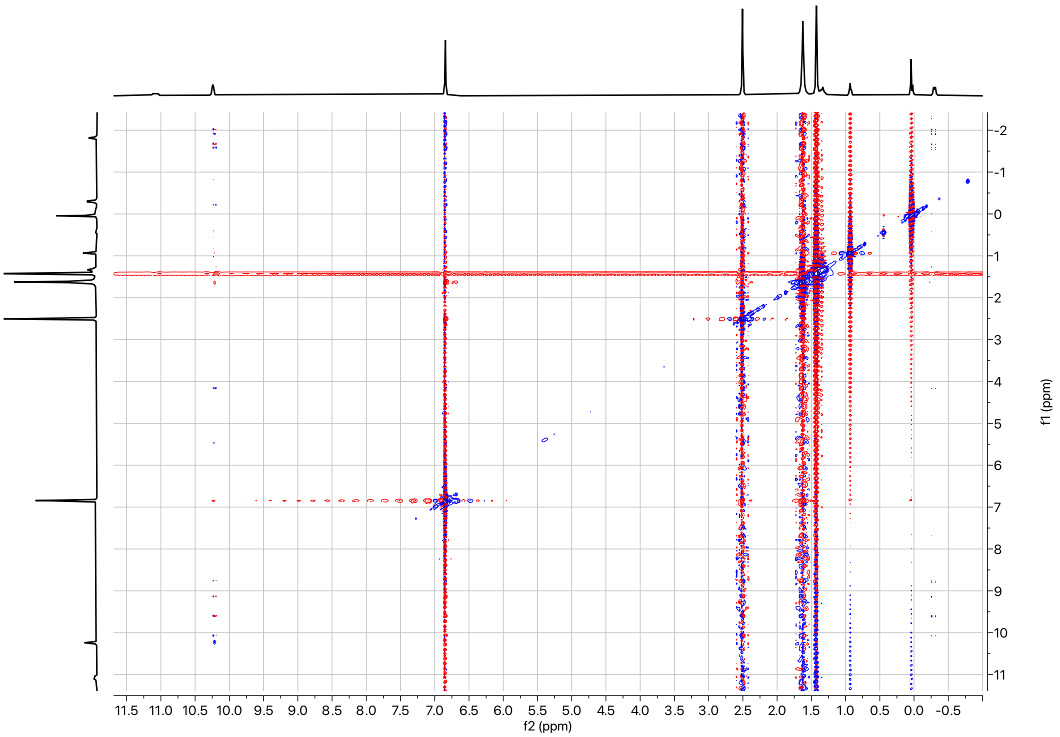

Figure S3. Representative EXSY spectrum of [(^{Mes}DI)Co(η^3-C_3H_5)(μ-N_2)]$_2$ in cyclohexane-d_{12}. No crosspeaks were observed between syn (10.14 ppm) and anti (-0.36 ppm) allyl proton resonances, providing evidence against η^1, η^3 coordination mode interconversion.

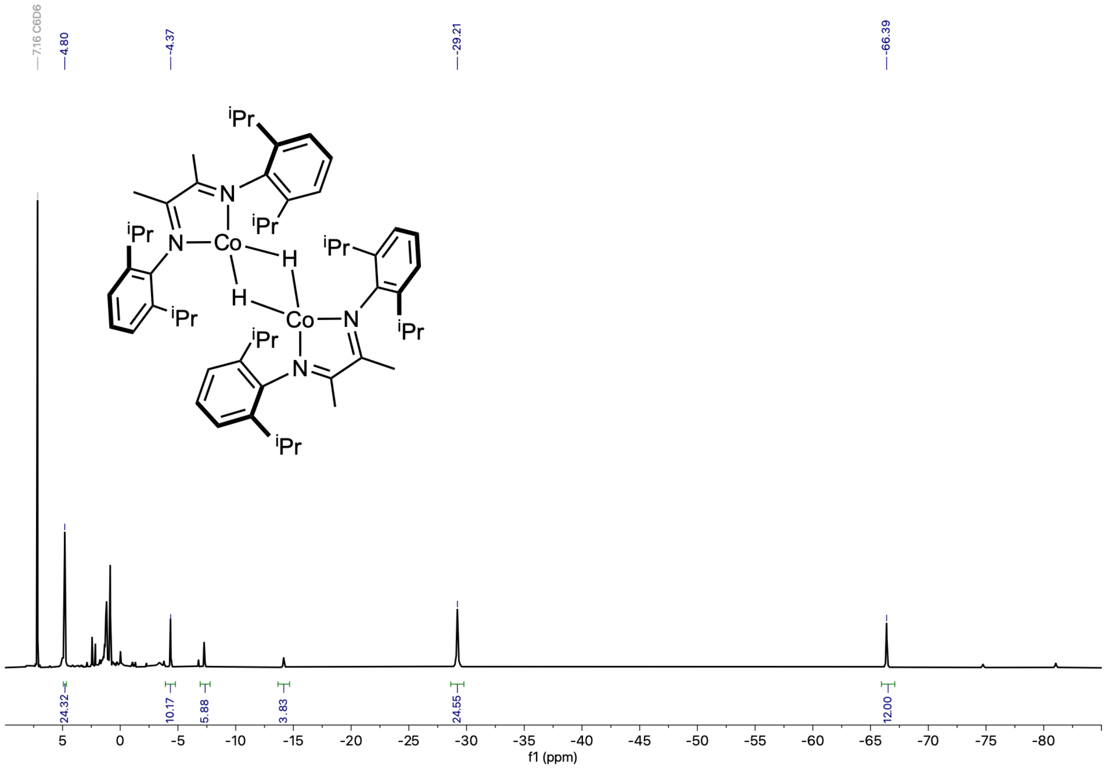

Figure S4. Representative ^{1}H NMR spectrum of [(^{iPr}DI)Co(H)]$_2$ (**[Co-H]$_2$**) in benzene-d_6.

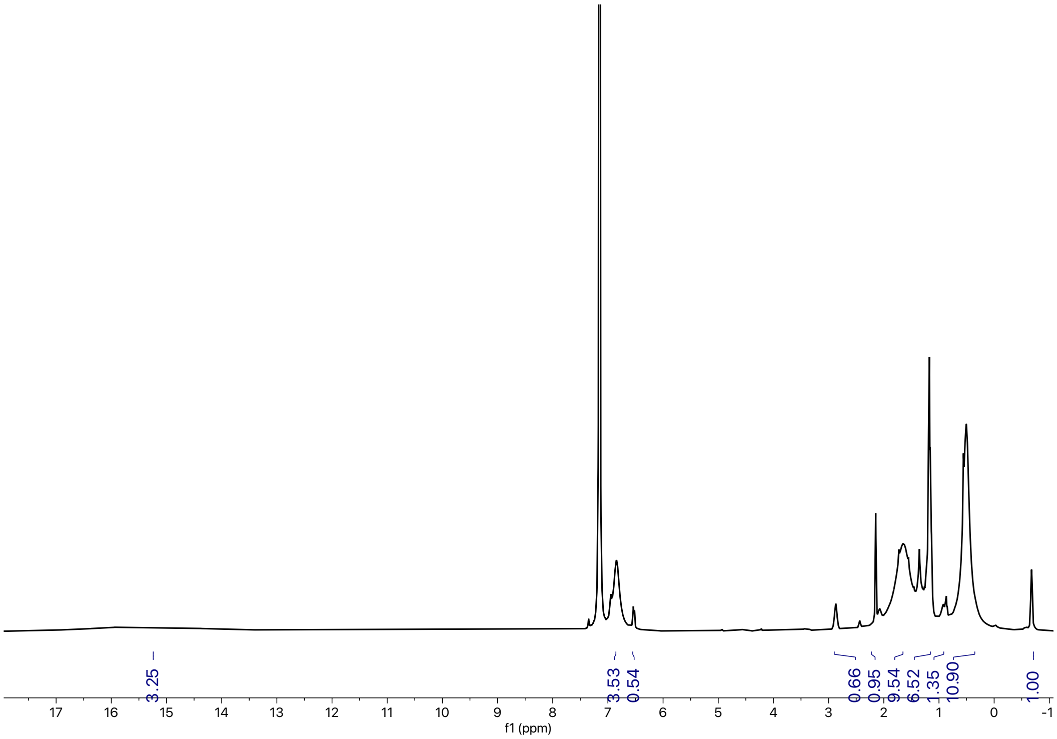

Figure S5. Representative ^1H NMR spectrum of the mixture of (^{iPr}DI)Co(η^6-C_6H_6) and [(^{iPr}DI)Co]$_2$ (examples of **Co(0)**).

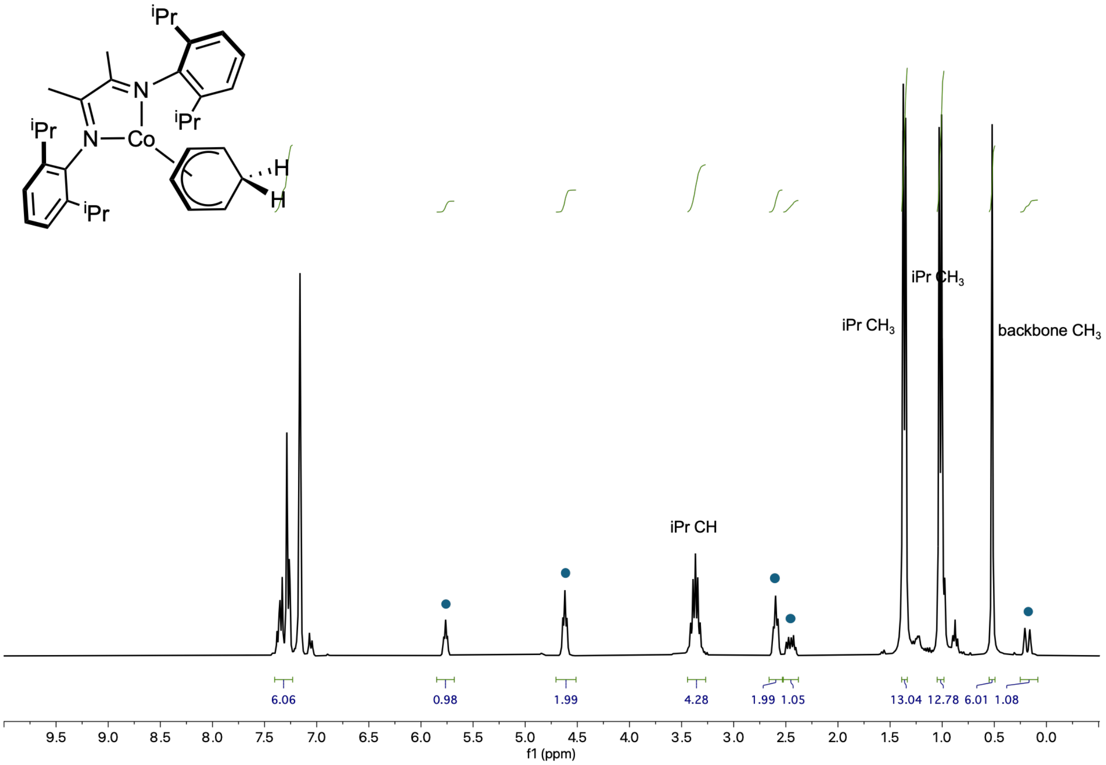

Figure S6. Representative ^1H NMR spectrum of (^{iPr}DI)Co(η^5-C_6H_7) (**Co1**) in benzene-d_6. Blue circles indicate cyclohexadienyl ligand resonances.

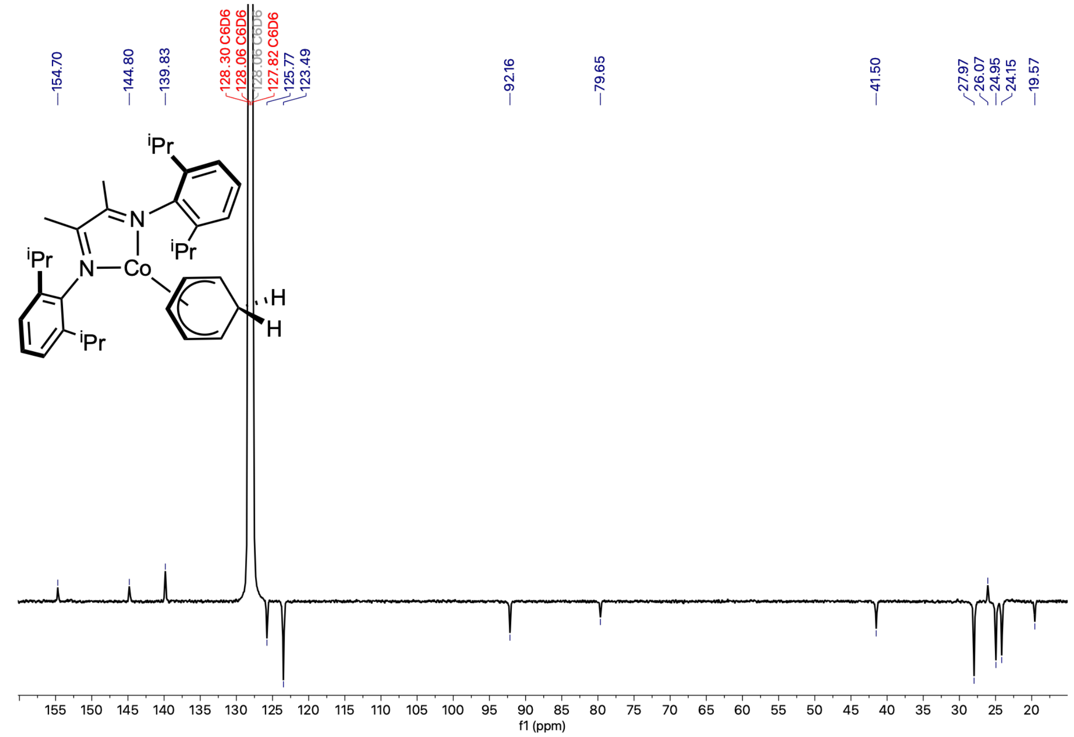

Figure S7. Representative APT ^{13}C NMR spectrum of (^{iPr}DI)Co(η^5-C_6H_7) (**Co1**) in benzene-d_6.

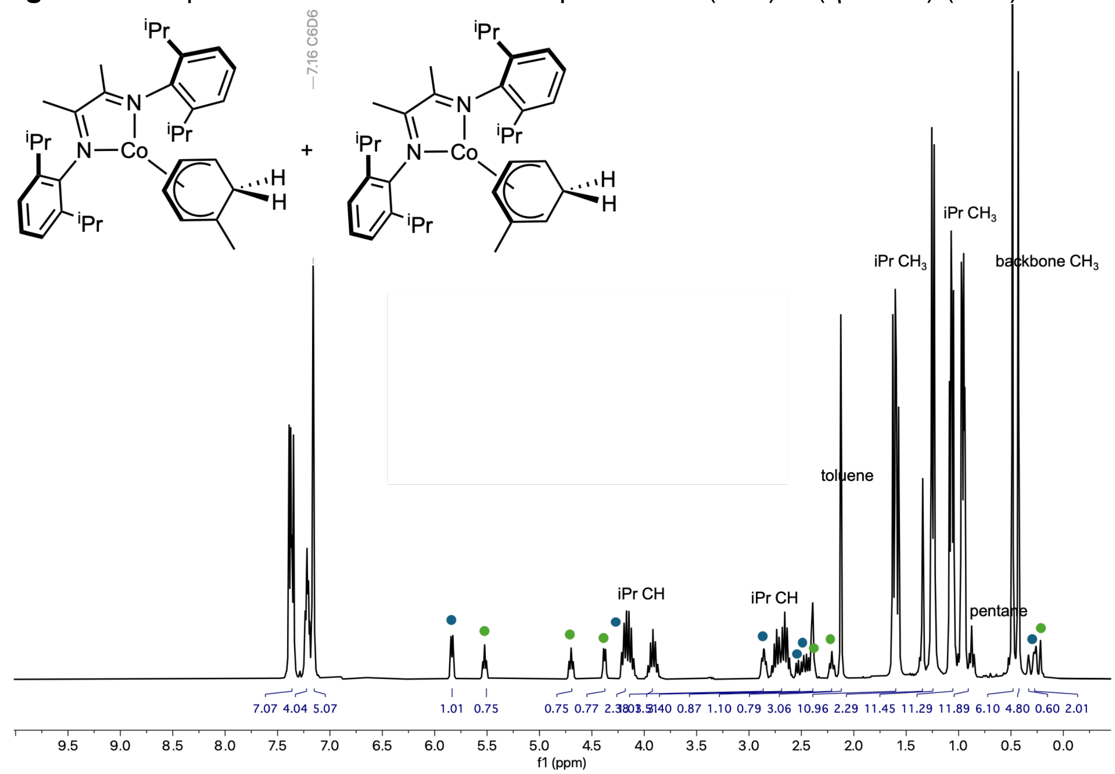

Figure S8. Representative ^1H NMR spectrum of (^{iPr}DI)Co(η^5-MeC_6H_7) (**Co2**) in benzene-d_6. Blue and green circles indicate cyclohexadienyl ligand resonances for each insertion isomer.

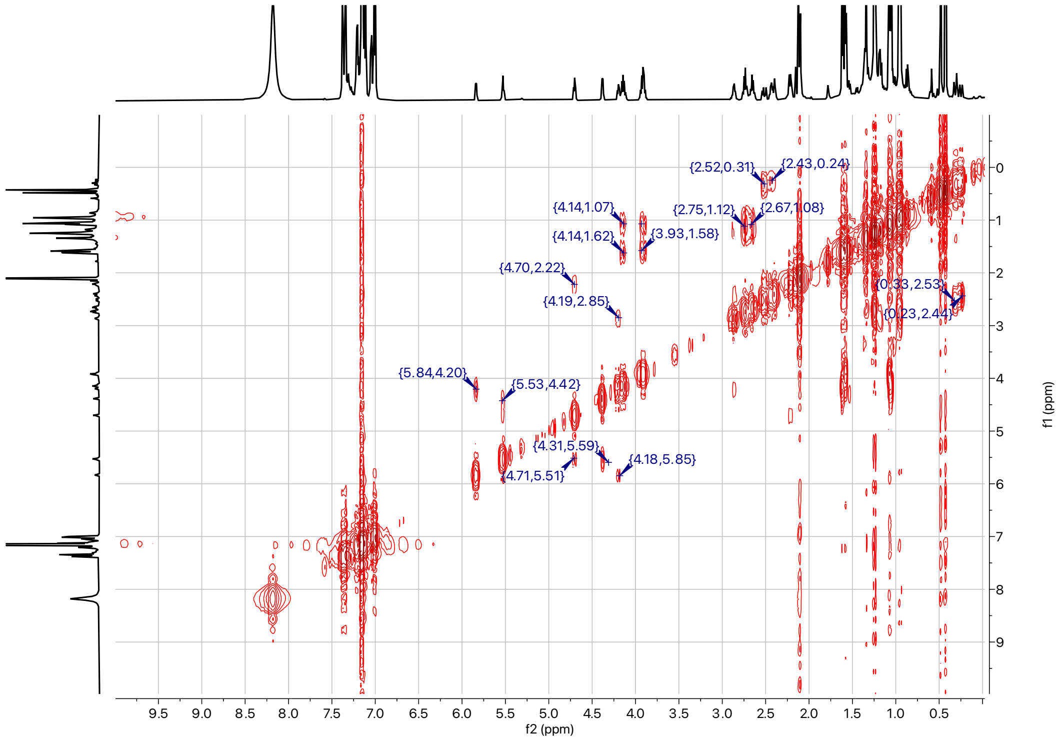

Figure S9. Representative COSY NMR spectrum of (^{iPr}DI)Co(η^5-MeC$_6$H$_7$) (**Co2**) containing 1,3,5-tristrifluoromethylbenzene in benzene-d_6.

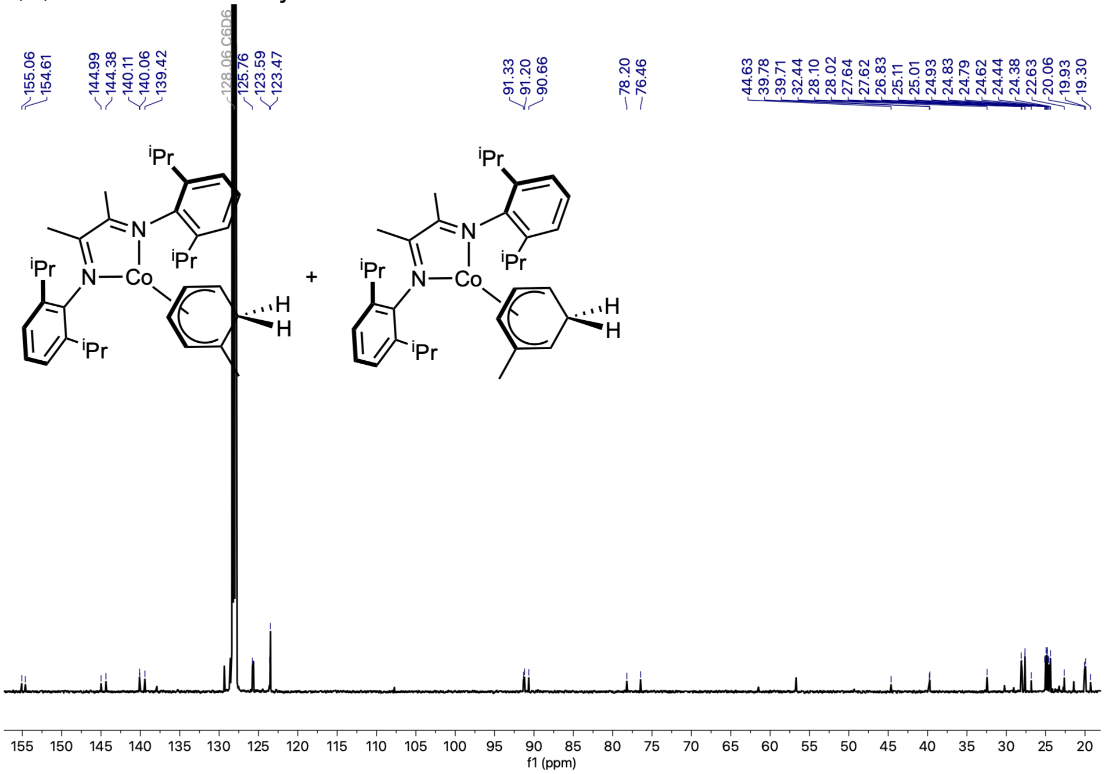

Figure S10. Representative ^{13}C NMR spectrum of (^{iPr}DI)Co(η^5-MeC$_6$H$_7$) (**Co2**) in benzene-d_6.

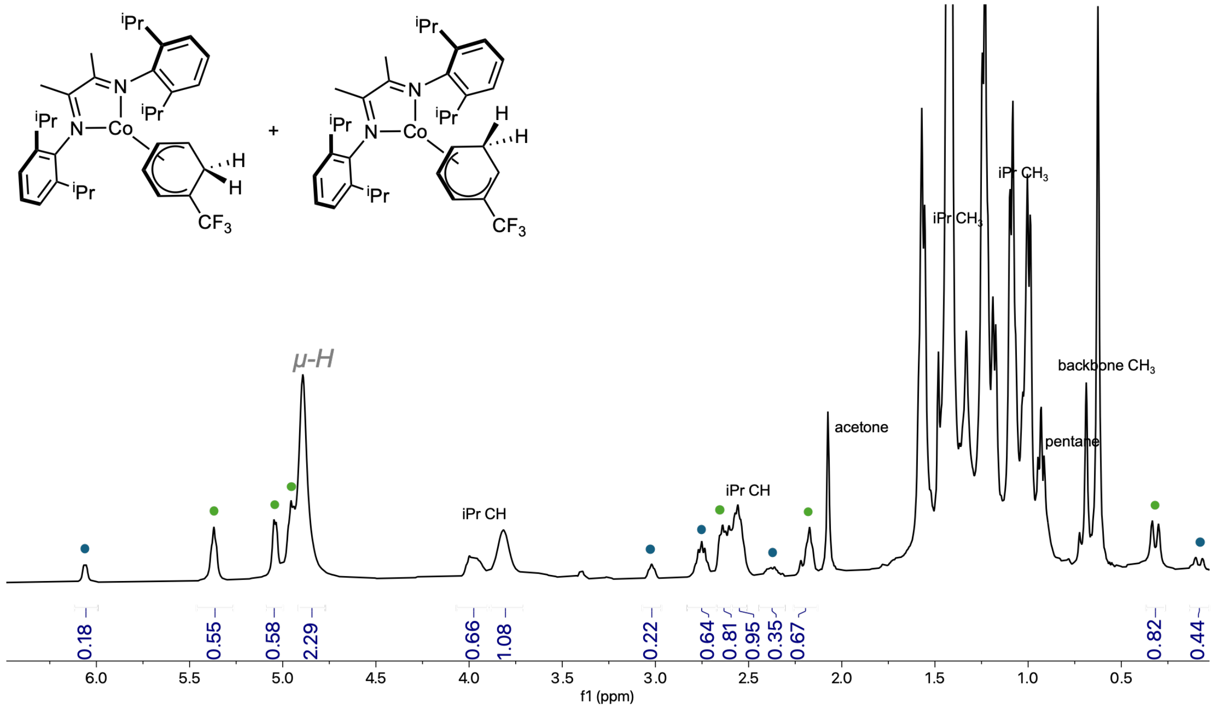

Figure S11. Representative ^{1}H NMR spectrum of (^{iPr}DI)Co(η^5-$CF_3C_6H_7$) (**Co3**) containing 8% **[Co-H]$_2$** and 1,3,5-tristrifluoromethylbenzene (~8 ppm) in cyclohexane-*d*$_{12}$. Blue and green circles indicate cyclohexadienyl ligand resonances for each insertion isomer.

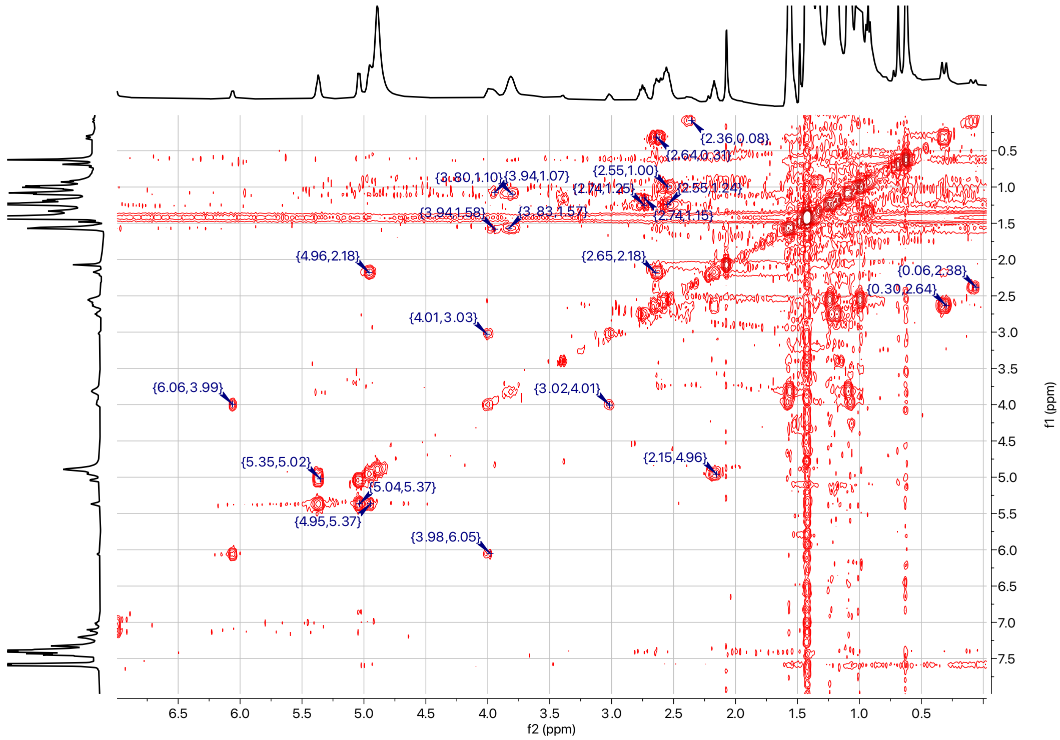

Figure S12. Representative COSY NMR spectrum of (^{iPr}DI)Co(η^5-$CF_3C_6H_7$) (**Co3**) in cyclohexane-*d*$_{12}$.

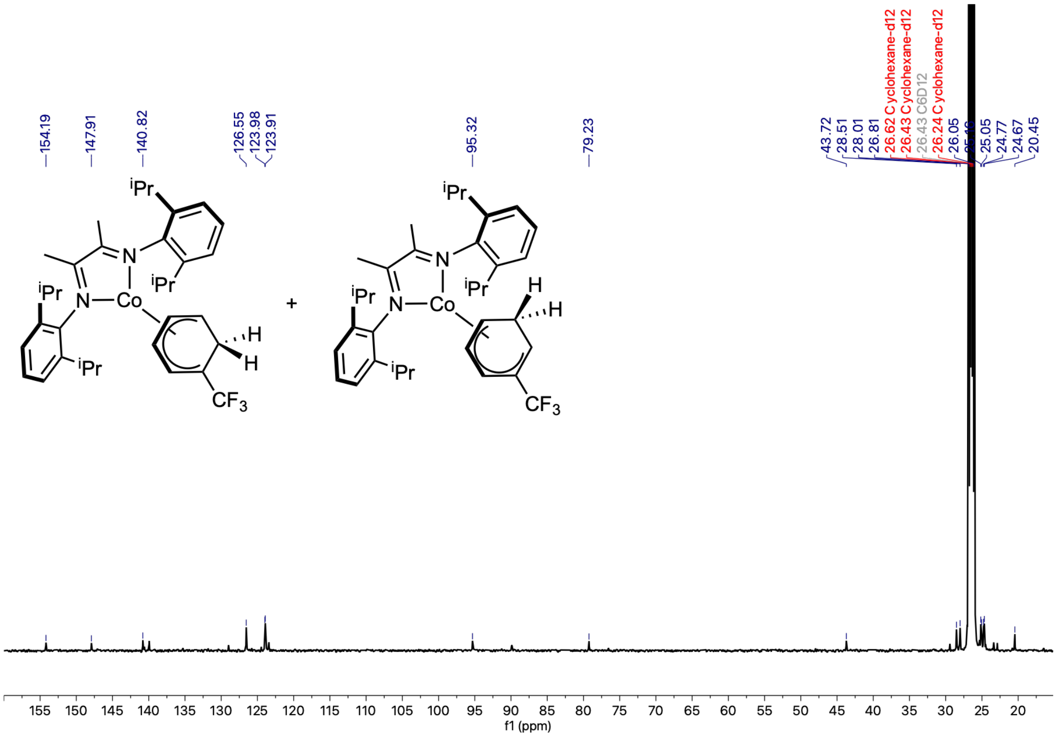

Figure S13. Representative ^{13}C NMR spectrum of (^{iPr}DI)Co(η^5-$CF_3C_6H_7$) (**Co3**) in cyclohexane-d_{12}.

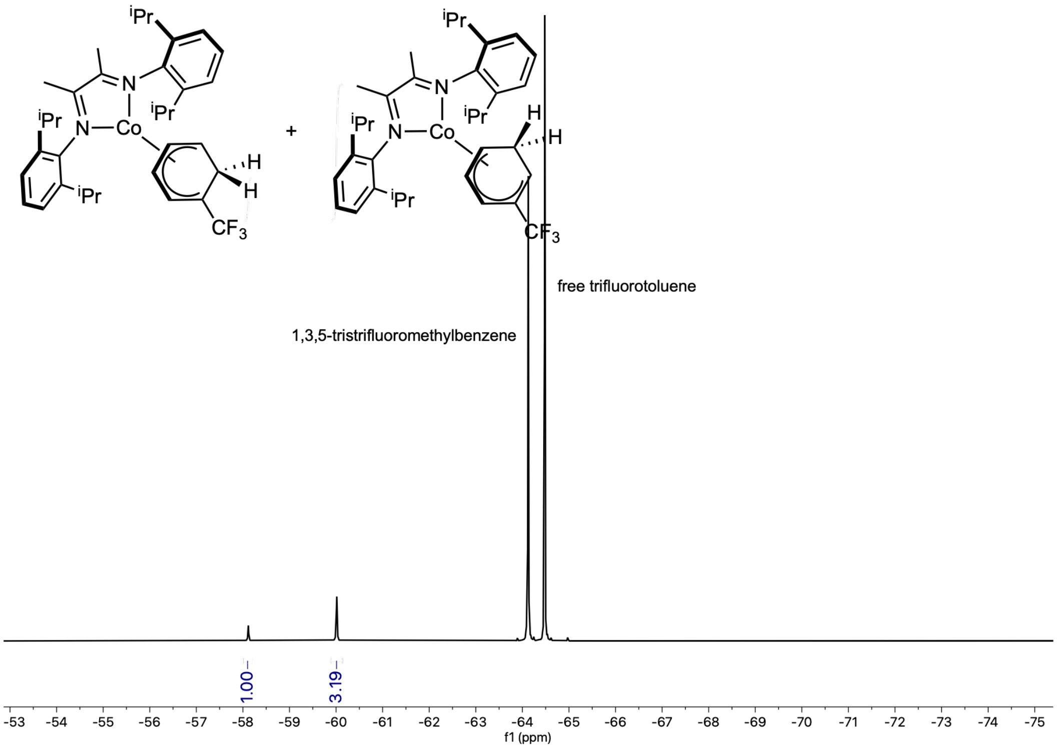

Figure S14. Representative ^{19}F NMR spectrum of (^{iPr}DI)Co(η^5-$CF_3C_6H_7$) (**Co3**) and 1,3,5-tristrifluoromethylbenzene in cyclohexane-d_{12}.

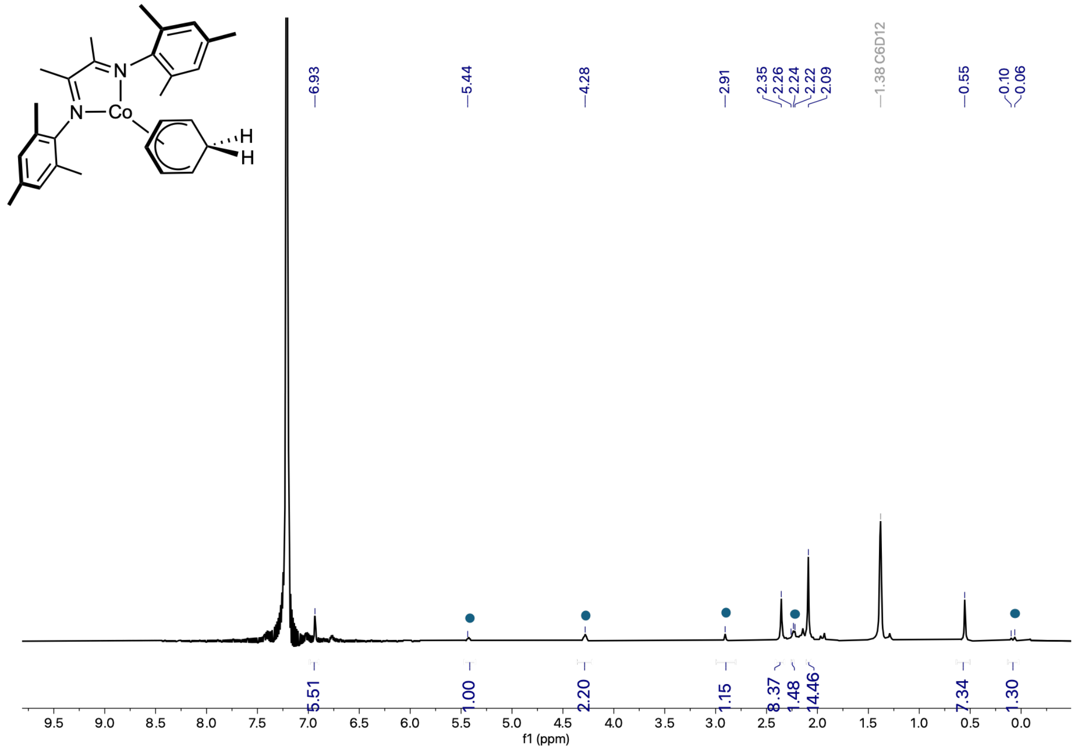

Figure S15. Representative ^{1}H NMR spectrum of (^{Mes}DI)Co(η^5-C_6H_7) (**Co4**) in cyclohexane-d_{12} with excess C_6H_6. Blue circles indicate cyclohexadienyl ligand resonances.

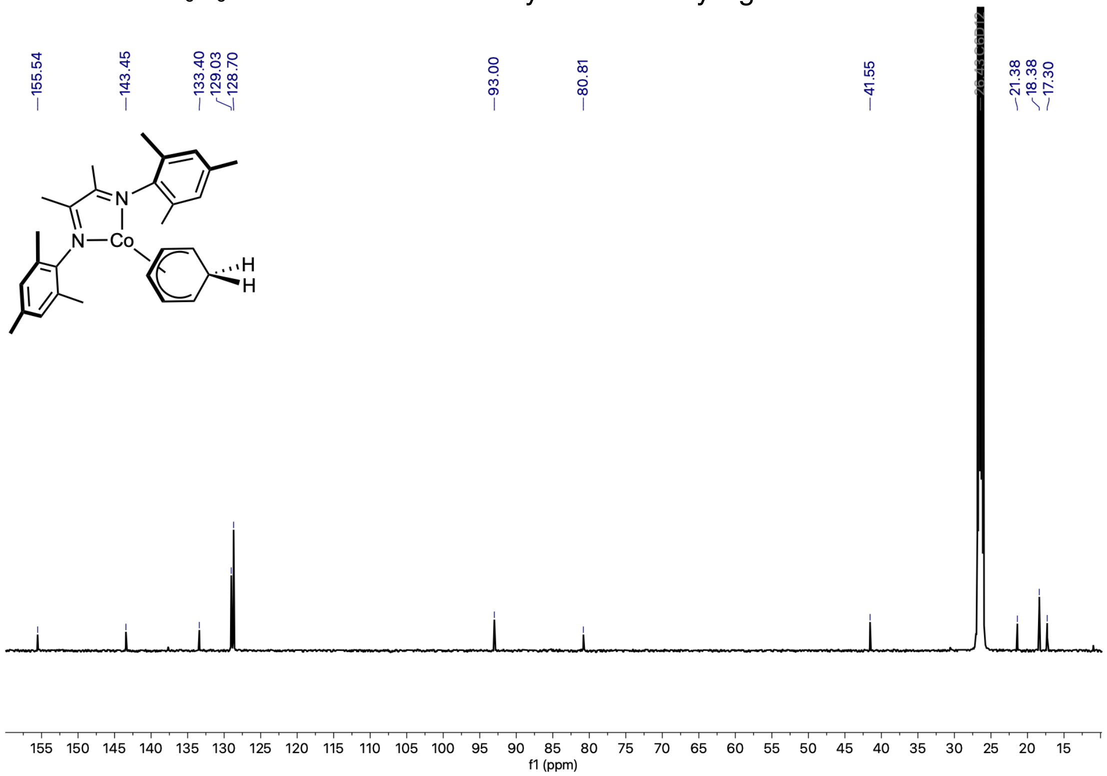

Figure S16. Representative ^{13}C NMR spectrum of (^{Mes}DI)Co(η^5-C_6H_7) (**Co4**) in cyclohexane-d_{12}.

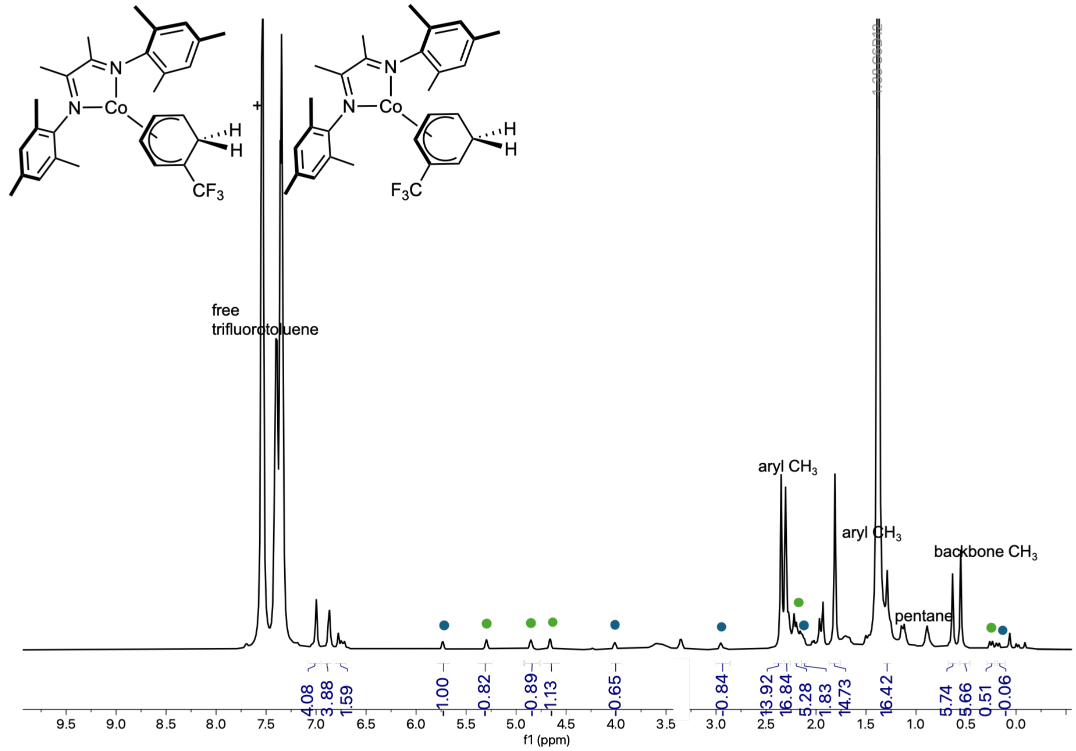

Figure S17. Representative ^{1}H NMR spectrum of (^{Mes}DI)Co(η^5-$CF_3C_6H_7$) (**Co5**) in cyclohexane-*d*$_{12}$. Blue and green circles indicate cyclohexadienyl ligand resonances for each insertion isomer.

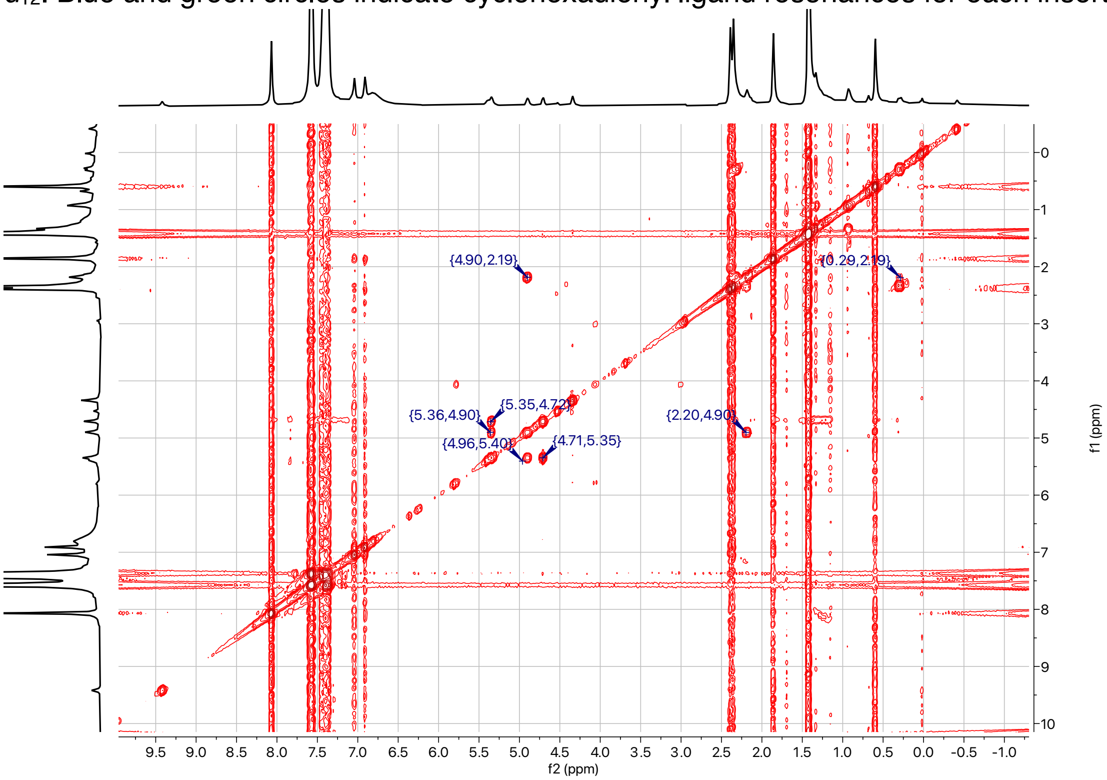

Figure S18. Representative COSY NMR spectrum of (^{Mes}DI)Co(η^5-$CF_3C_6H_7$) (**Co5**) containing 1,3,5-tristrifluoromethylbenzene (~8 ppm) in cyclohexane-*d*$_{12}$.

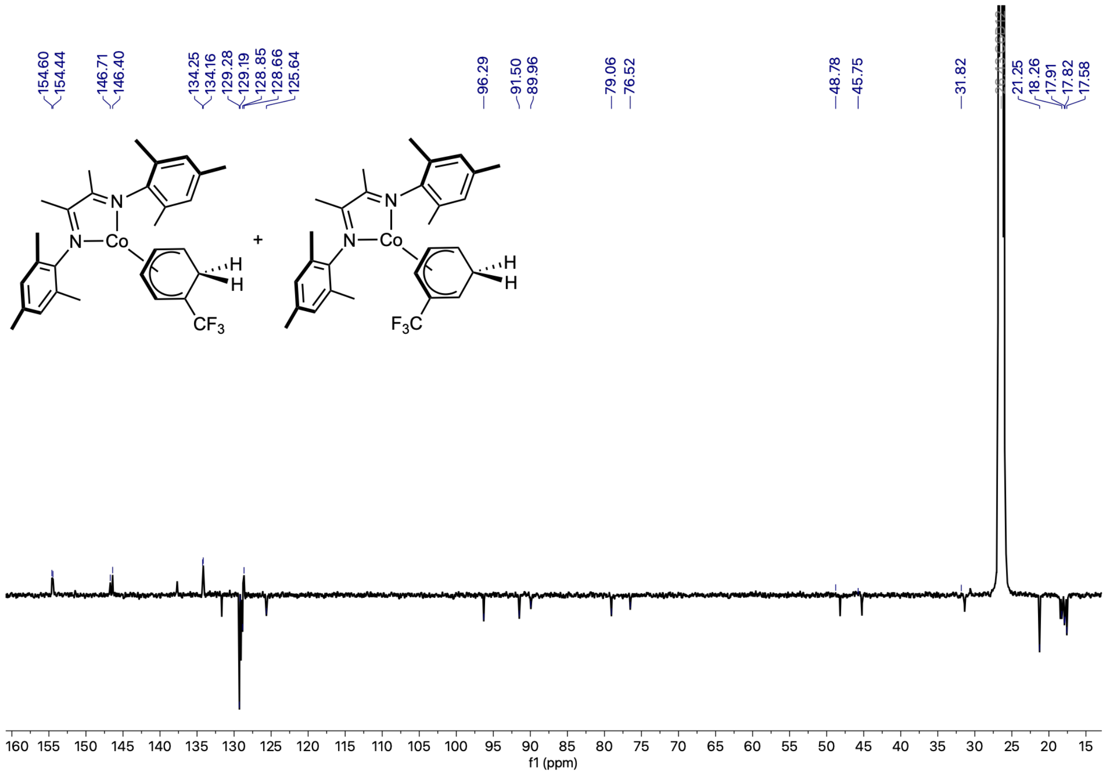

Figure S19. Representative ^{13}C APT NMR spectrum of (^{Mes}DI)Co(η^5-$CF_3C_6H_7$) (**Co5**) in cyclohexane-d_{12}.

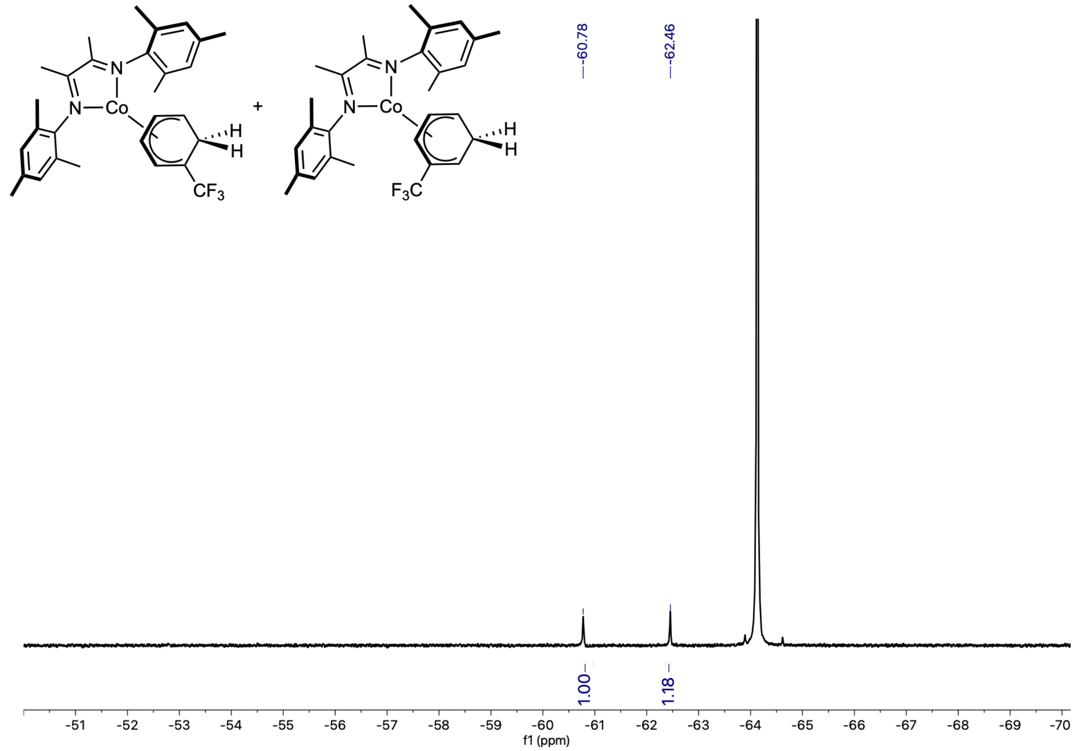

Figure S20. Representative ^{19}F NMR spectrum of (^{Mes}DI)Co(η^5-$CF_3C_6H_7$) (**Co5**) containing free α,α,α-trifluorotoluene in cyclohexane-d_{12}.

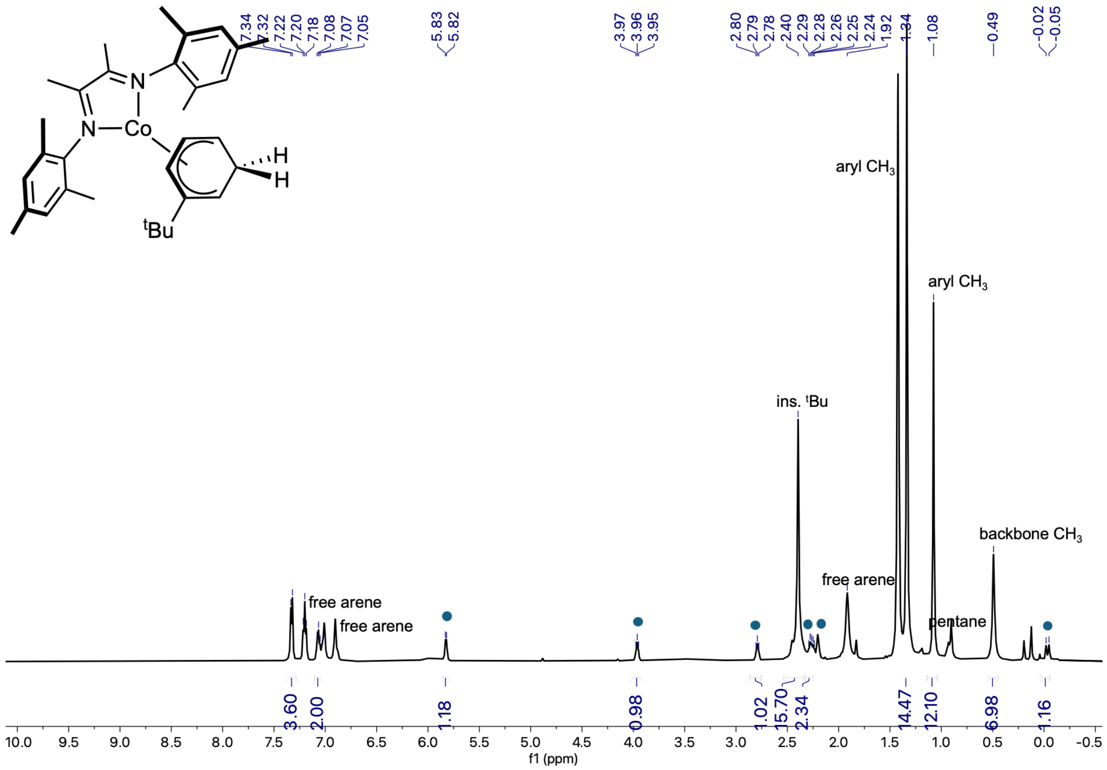

Figure S21. Representative ^1H NMR spectrum of (^{Mes}DI)Co(η^5-$^tBuC_6H_7$) (**Co6**) in cyclohexane-d_{12}. Blue circles indicate cyclohexadienyl ligand resonances.

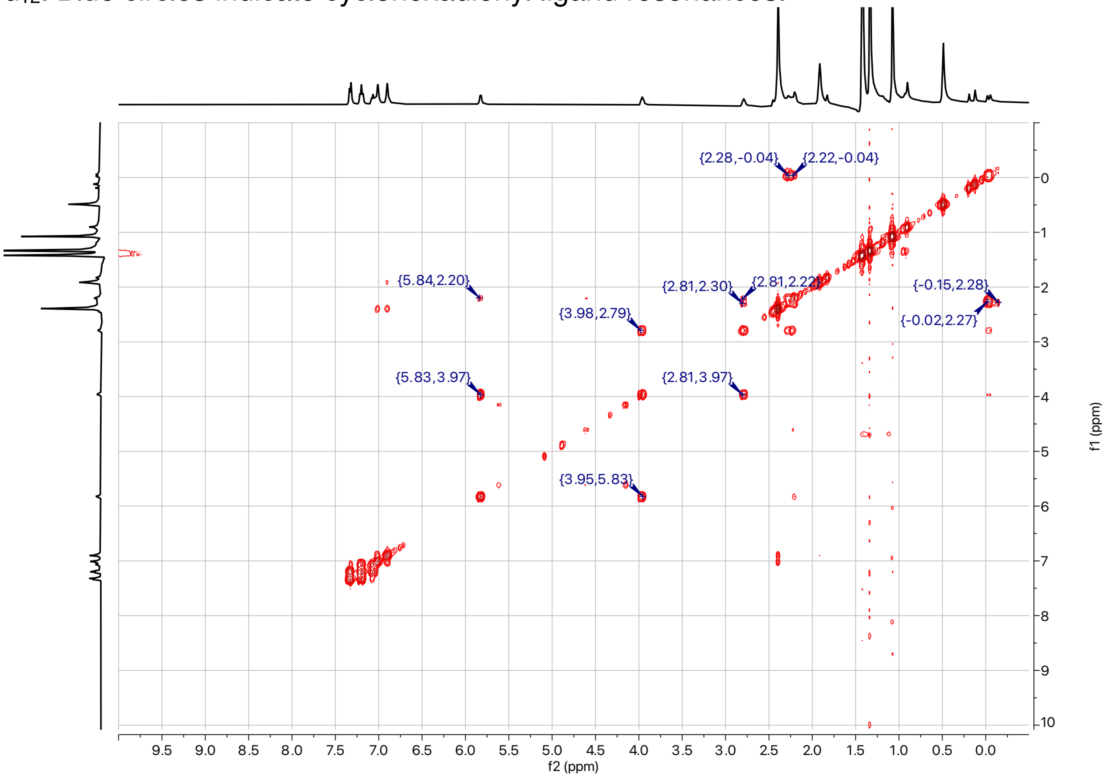

Figure S22. Representative COSY NMR spectrum of (^{Mes}DI)Co(η^5-$^tBuC_6H_7$) (**Co6**) in cyclohexane-d_{12}.

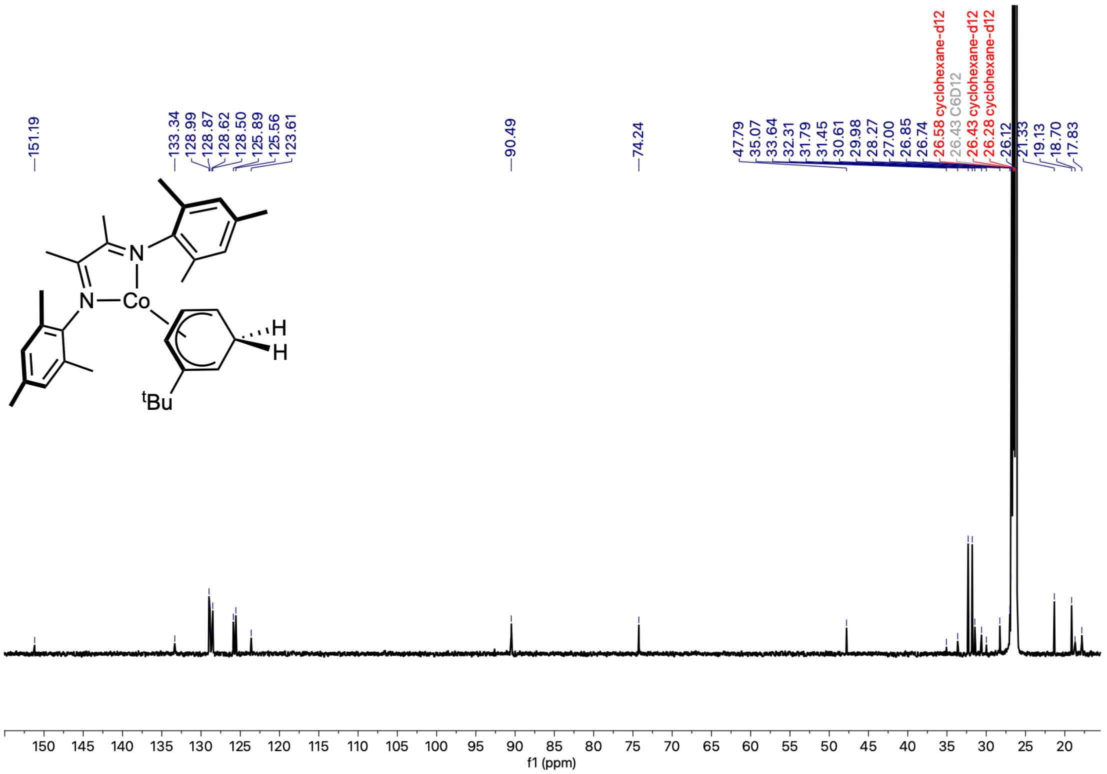

Figure S23. Representative ^{13}C NMR spectrum of (^{Mes}DI)Co(η^5-$^tBuC_6H_7$) (**Co6**) in cyclohexane-*d*$_{12}$.

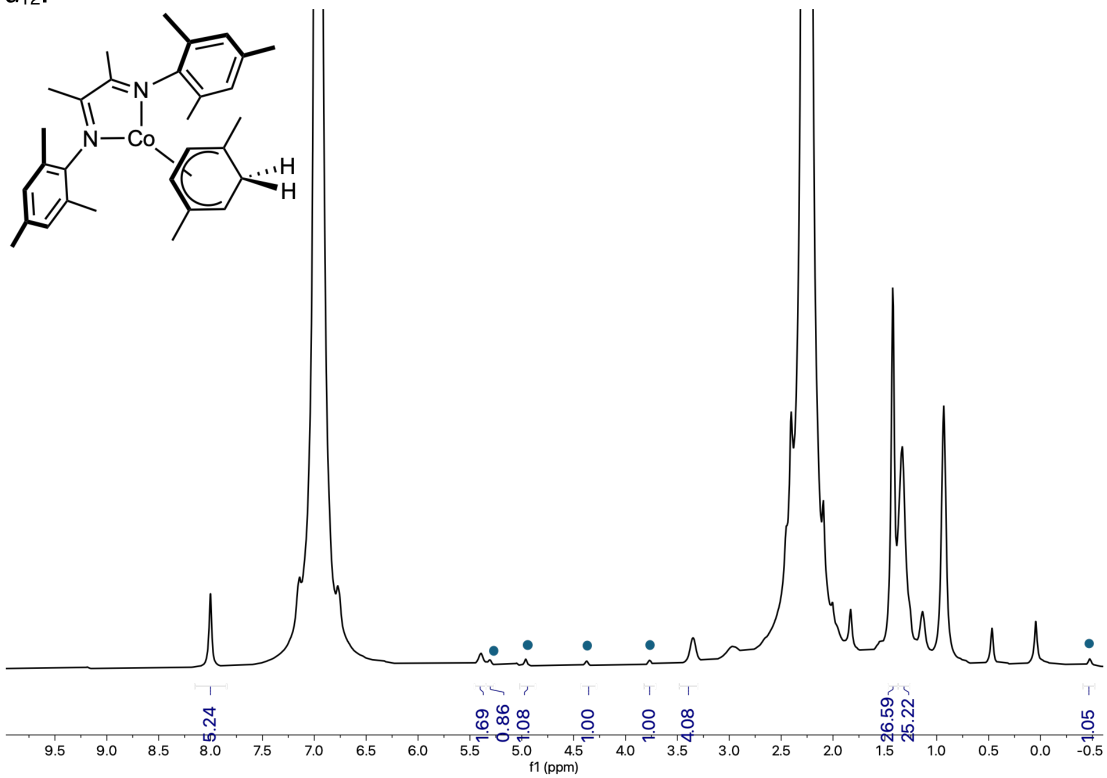

Figure S24. Representative ^1H NMR spectrum of (^{Mes}DI)Co(*p*-xylene) (**Co7**) with 1,3,5-tristrifluoromethylbenzene in cyclohexane-*d*$_{12}$. Blue circles indicate cyclohexadienyl ligand resonances. Note: COSY experiments lacked clear correlation between proton resonances.

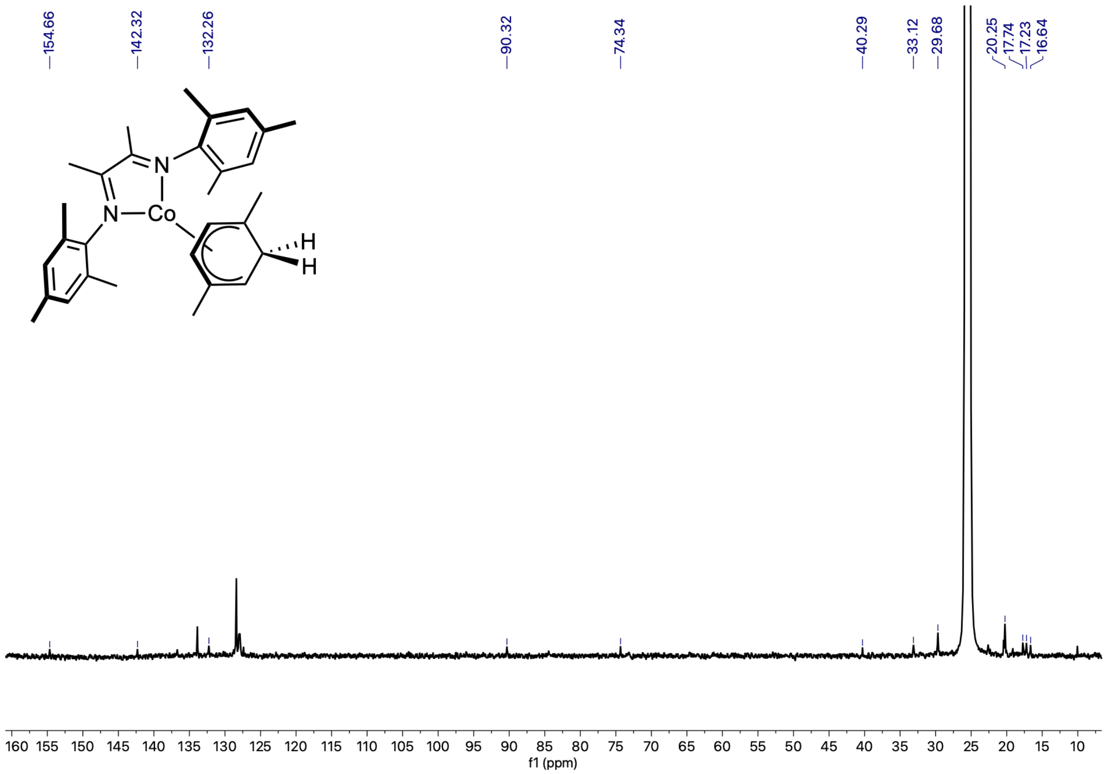

Figure S25. Representative ^{13}C NMR spectrum of (^{Mes}DI)Co(*p*-xylene) (**Co7**) in cyclohexane-d_{12}.

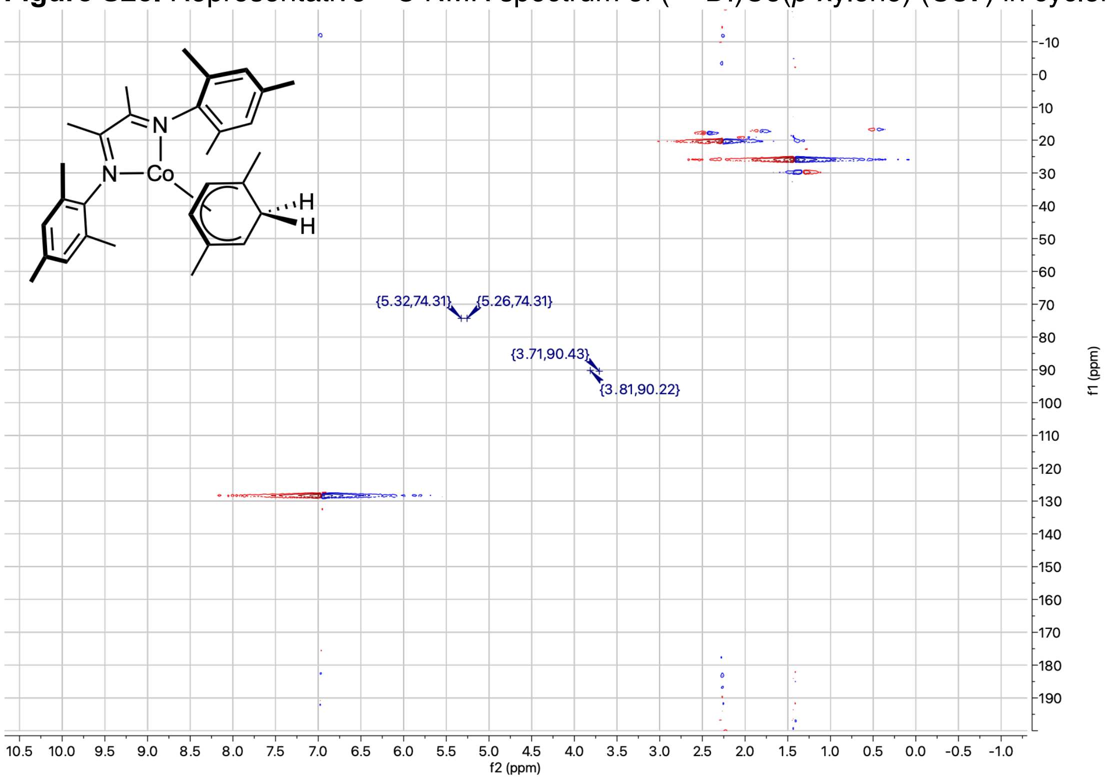

Figure S26. Representative HSQC NMR spectrum of (^{Mes}DI)Co(*p*-xylene) (**Co7**) in cyclohexane-d_{12}. This allowed for identification of cyclohexadienyl resonances and allowed for quantification of insertion yield by ^{1}H NMR spectroscopy.

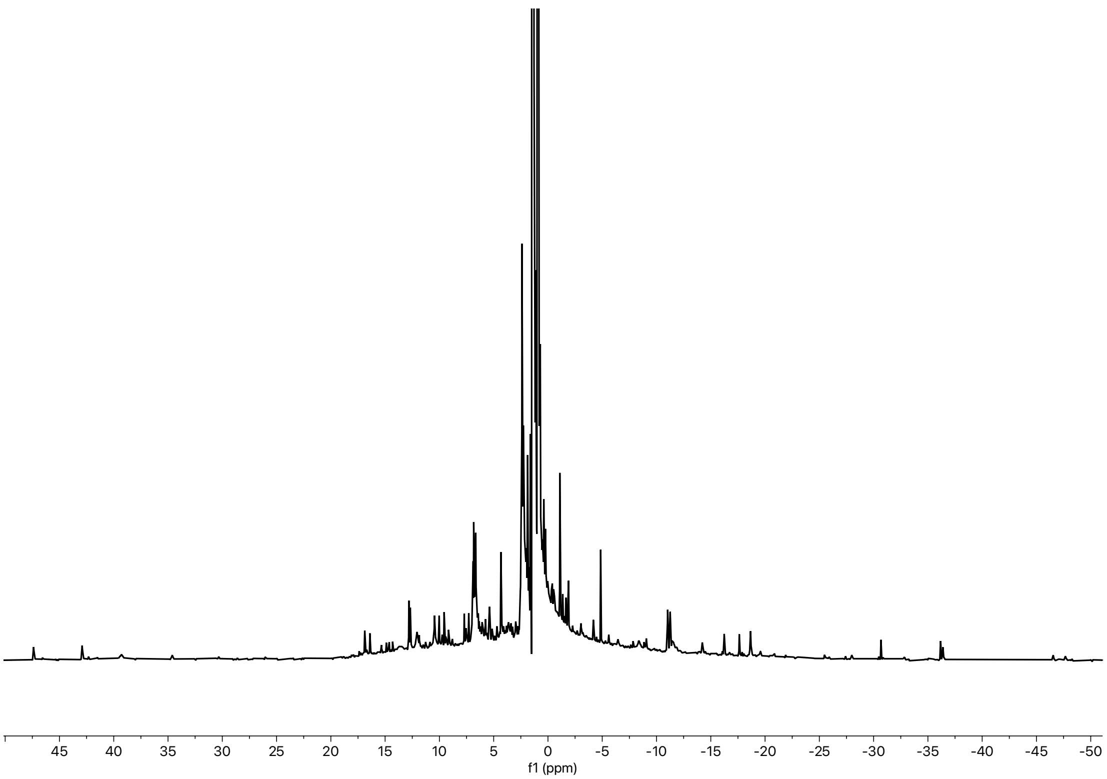

Figure S27. ^{1}H NMR spectrum resulting from exposing [(^{Mes}DI)Co(η^3-C_3H_5)(μ-N_2)]$_2$ to 1 atm H_2 in cyclohexane-d_{12}.

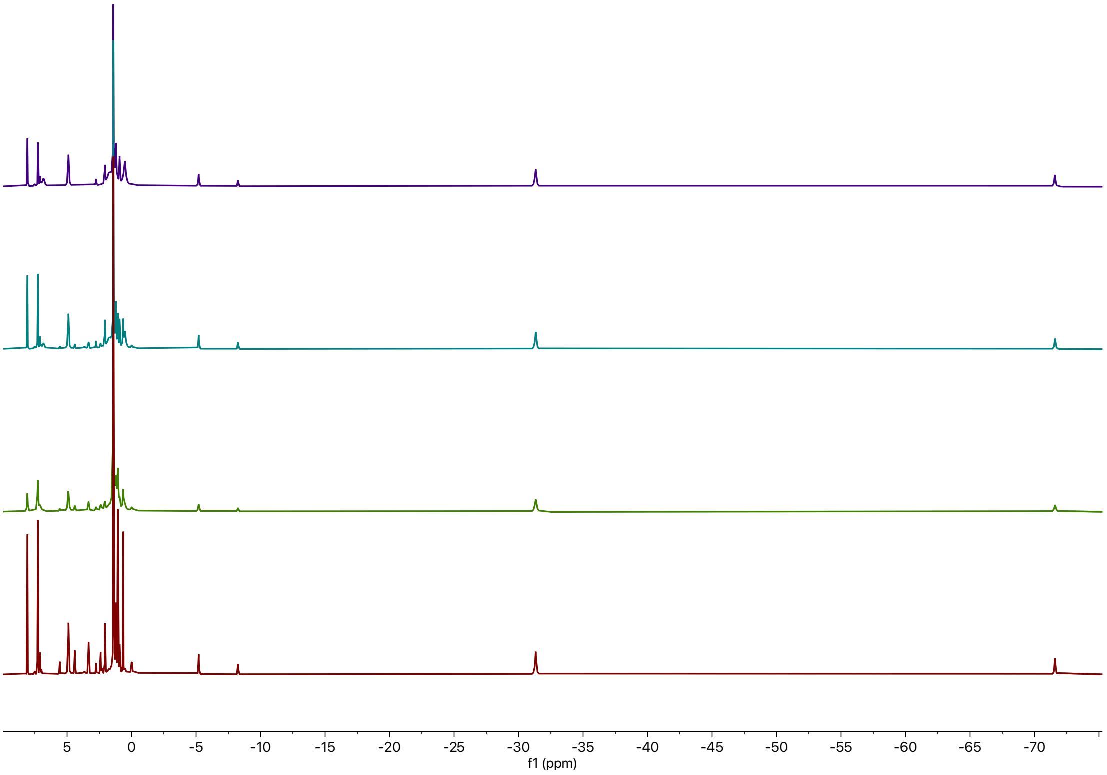

Figure S28. ^{1}H NMR spectra of (^{iPr}DI)Co(η^5-C_6H_7) in cyclohexane-d_{12} over 72 hours. Bottom to top: 30 min at 25 °C, 6 h at 25 °C, 24 h at 25°C, 72 h at 25 °C.

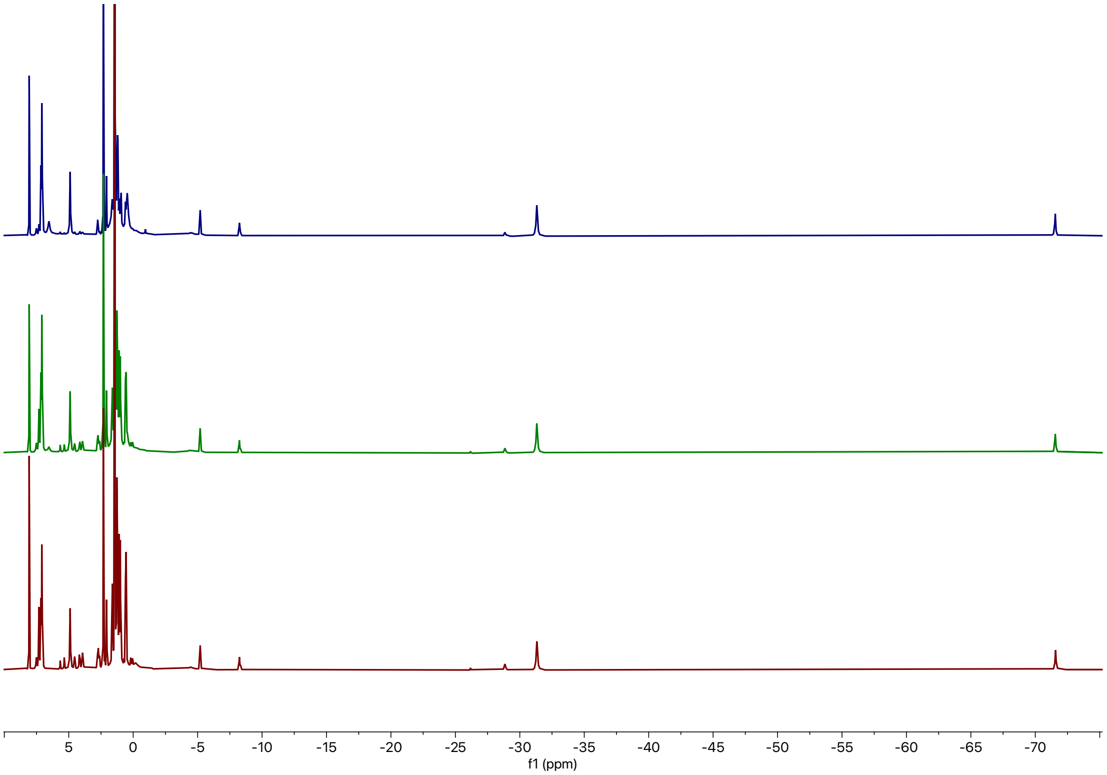

Figure S29. ^{1}H NMR spectra of $(^{iPr}DI)Co(\eta^5\text{-}MeC_6H_7)$ in cyclohexane-d_{12} over 72 hours. Bottom to top: 30 min at 25 °C, 24 h at 25 °C, 72 h at 25 °C.

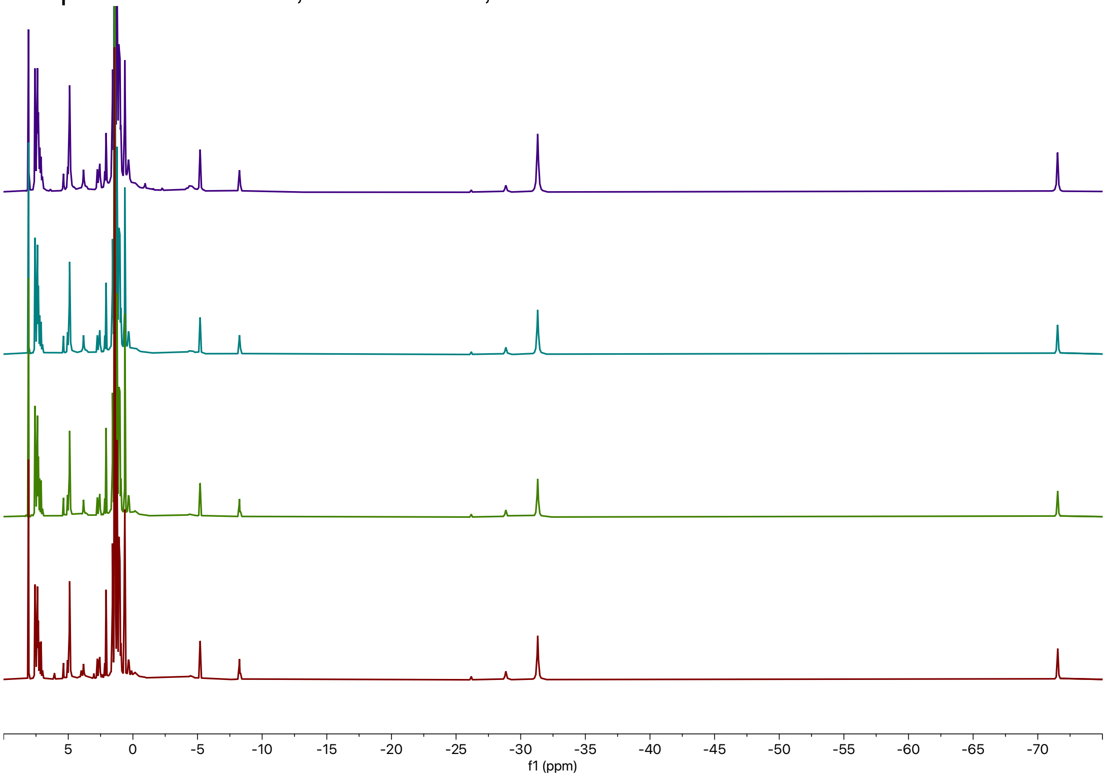

Figure S30. ^{1}H NMR spectra of $(^{iPr}DI)Co(\eta^5\text{-}CF_3C_6H_7)$ in cyclohexane-d_{12} over 72 hours. Bottom to top: 30 min at 25 °C, 6 h at 25 °C, 24 h at 25 °C, 72 h at 25 °C.

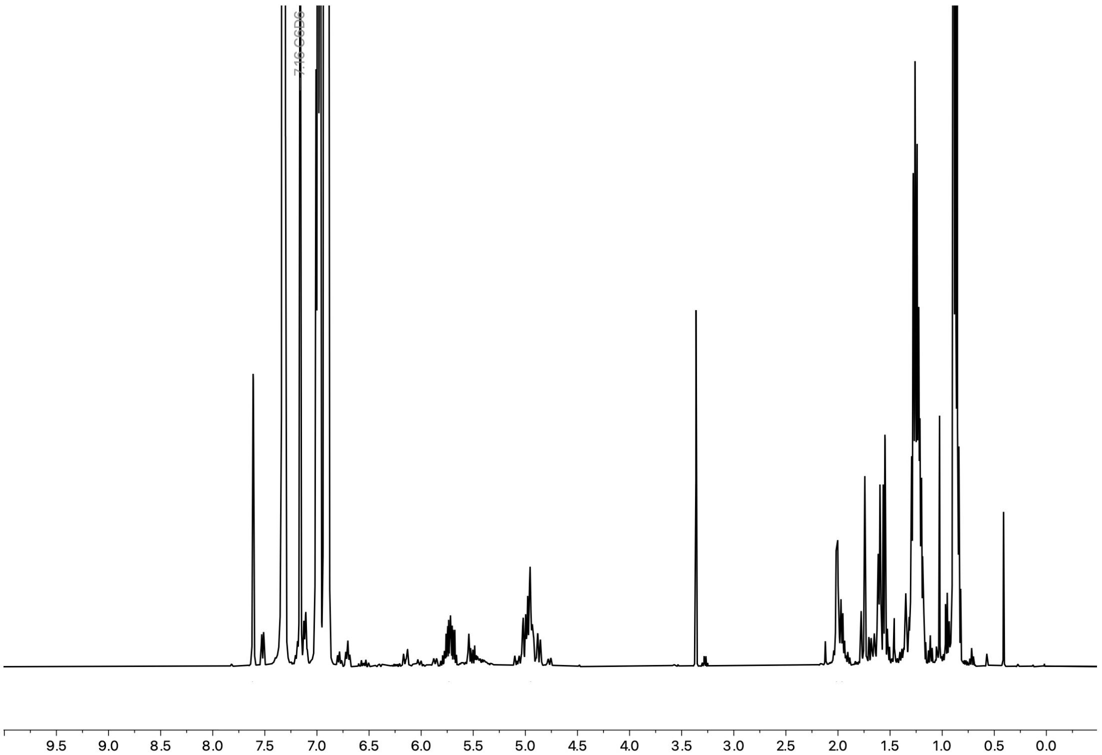

Figure S31. ^{1}H NMR spectrum of volatile products resulting from the protonation of (^{iPr}DI)Co(η^5-$CF_3C_6H_7$) with benzoic acid in benzene-d_6.

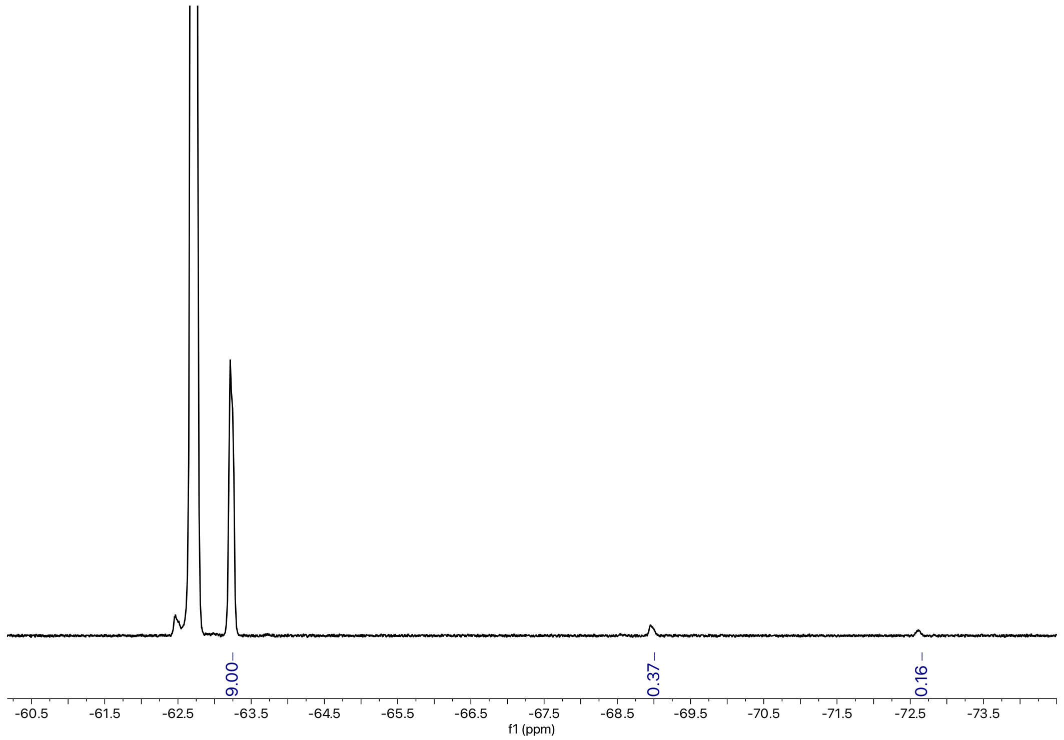

Figure S32. ^{19}F NMR spectrum of volatile products resulting from the protonation of (^{iPr}DI)Co(η^5-$CF_3C_6H_7$) with benzoic acid in benzene-d_6. The resonance around -72.5 ppm is consistent with that reported for the C(sp^3)-CF_3-subsituted 3,5-cyclohexadiene.[6]

VI. Crystallographic Data

Program(s) used to solve structure: SHELXT 2014/4 (Sheldrick, 2014); program(s) used to refine structure: *SHELXL* 2018/3 (Sheldrick, 2015); molecular graphics: Olex2 1.5 (Dolomanov *et al.*, 2009); software used to prepare material for publication: Olex2 1.5 (Dolomanov *et al.*, 2009).

[(^{Mes}DI)Co(η^3-C_3H_5)(μ-N_2)]$_2$

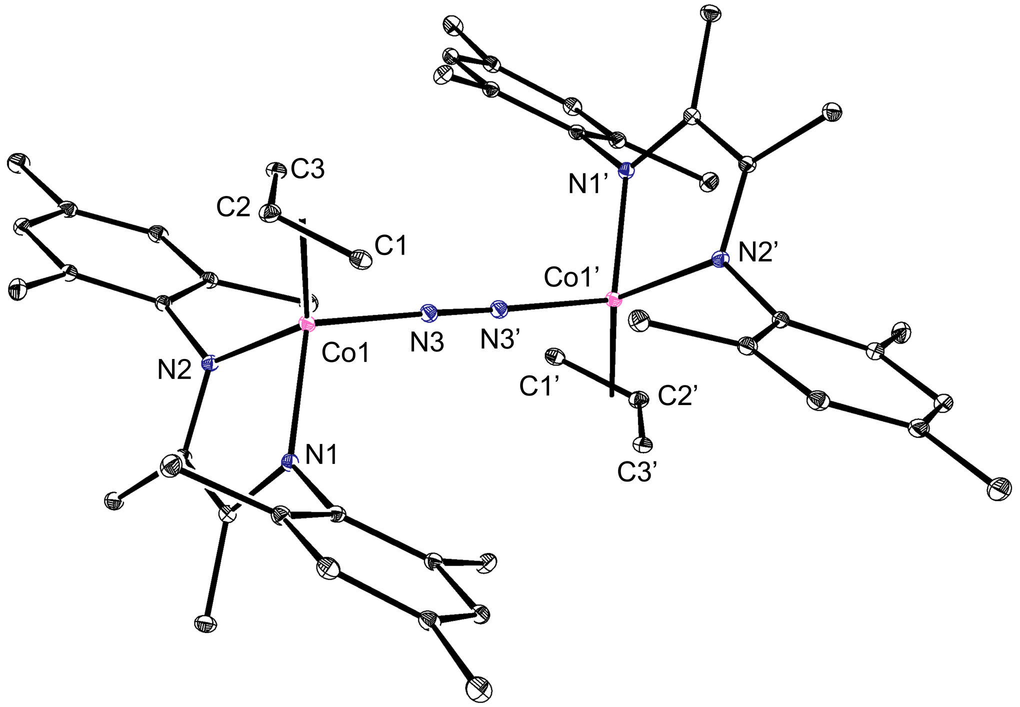

Computing details

Data collection: *CrysAlis PRO* 1.171.43.143a (Rigaku OD, 2024); cell refinement: *CrysAlis PRO* 1.171.43.143a (Rigaku OD, 2024); data reduction: *CrysAlis PRO* 1.171.43.143a (Rigaku OD, 2024); program(s) used to solve structure: SHELXT 2018/2 (Sheldrick, 2018); program(s) used to refine structure: *SHELXL* 2018/3 (Sheldrick, 2015); molecular graphics: Olex2 1.5 (Dolomanov *et al.*, 2009); software used to prepare material for publication: Olex2 1.5 (Dolomanov *et al.*, 2009).

(klm13_auto)

Crystal data

$C_{25}H_{33}CoN_3$	$F(000) = 924$
$M_r = 434.47$	$D_x = 1.286$ Mg m^{-3}
Monoclinic, $P2_1/n$	Cu $K\alpha$ radiation, $\lambda = 1.54184$ Å
$a = 12.3692$ (2) Å	Cell parameters from 14126 reflections
$b = 13.3561$ (2) Å	$\theta = 4.6–73.7°$
$c = 14.2917$ (2) Å	$\mu = 6.09$ mm^{-1}
$\beta = 108.0799$ (19)°	$T = 100$ K
$V = 2244.48$ (7) Å^3	Plate, orange
$Z = 4$	0.16 × 0.12 × 0.06 mm

Data collection

XtaLAB Synergy, Dualflex, HyPix-Arc 150 diffractometer	4548 independent reflections
Radiation source: micro-focus sealed X-ray tube, PhotonJet (Cu) X-ray Source	4188 reflections with $I > 2\sigma(I)$
Mirror monochromator	$R_{int} = 0.039$
Detector resolution: 10.0000 pixels mm^{-1}	$\theta_{max} = 75.8°$, $\theta_{min} = 4.6°$
ω scans	$h = -14\rightarrow15$
Absorption correction: multi-scan *CrysAlis PRO* 1.171.43.143a (Rigaku Oxford Diffraction, 2024) Empirical absorption correction using spherical harmonics, implemented in SCALE3 ABSPACK scaling algorithm.	$k = -16\rightarrow16$
$T_{min} = 0.590$, $T_{max} = 1.000$	$l = -17\rightarrow16$
36520 measured reflections	

Refinement

Refinement on F^2	0 restraints
Least-squares matrix: full	Hydrogen site location: inferred from neighbouring sites
$R[F^2 > 2\sigma(F^2)] = 0.045$	H-atom parameters constrained
$wR(F^2) = 0.099$	$w = 1/[\sigma^2(F_o^2) + (0.0369P)^2 + 2.2631P]$ where $P = (F_o^2 + 2F_c^2)/3$
$S = 1.08$	$(\Delta/\sigma)_{max} = 0.001$
4548 reflections	$\Delta\rangle_{max} = 0.33$ e Å^{-3}
270 parameters	$\Delta\rangle_{min} = -0.37$ e Å^{-3}

Special details

Geometry. All esds (except the esd in the dihedral angle between two l.s. planes) are estimated using the full covariance matrix. The cell esds are taken into account individually in the estimation of esds in distances, angles and torsion angles; correlations between esds in cell parameters are only used when they are defined by crystal symmetry. An approximate (isotropic) treatment of cell esds is used for estimating esds involving l.s. planes.

(^{Mes}DI)$_2$Co

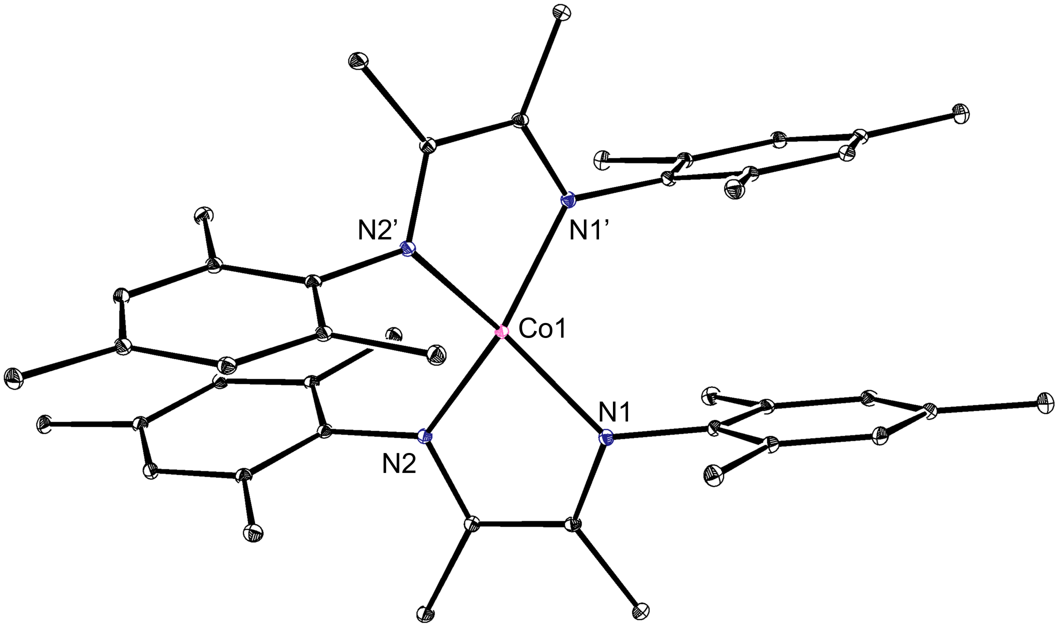

Computing details

Data collection: *CrysAlis PRO* 1.171.43.143a (Rigaku OD, 2024); cell refinement: *CrysAlis PRO* 1.171.43.143a (Rigaku OD, 2024); data reduction: *CrysAlis PRO* 1.171.43.143a (Rigaku OD, 2024); program(s) used to solve structure: SHELXT 2018/2 (Sheldrick, 2018); program(s) used to refine structure: *SHELXL* 2018/3 (Sheldrick, 2015); molecular graphics: Olex2 1.5 (Dolomanov *et al.*, 2009); software used to prepare material for publication: Olex2 1.5 (Dolomanov *et al.*, 2009).

(klm15_auto)

Crystal data

$C_{44}H_{56}CoN_4$	D_x = 1.233 Mg m^{-3}
M_r = 699.85	Cu $K\alpha$ radiation, λ = 1.54184 Å
Orthorhombic, *Fdd*2	Cell parameters from 7117 reflections
a = 25.5304 (3) Å	θ = 4.1–75.3°

b = 20.3513 (2) Å	μ = 3.83 mm^{-1}
c = 14.51252 (11) Å	*T* = 100 K
V = 7540.39 (12) Å^3	Plate, dark black
Z = 8	0.15 × 0.12 × 0.12 mm
F(000) = 3000	

Data collection

XtaLAB Synergy, Dualflex, HyPix-Arc 150 diffractometer	3201 independent reflections
Radiation source: micro-focus sealed X-ray tube, PhotonJet (Cu) X-ray Source	3108 reflections with $I > 2\sigma(I)$
Mirror monochromator	R_{int} = 0.019
Detector resolution: 10.0000 pixels mm^{-1}	θ_{max} = 75.7°, θ_{min} = 4.1°
ω scans	*h* = -29→31
Absorption correction: multi-scan *CrysAlis PRO* 1.171.43.143a (Rigaku Oxford Diffraction, 2024) Empirical absorption correction using spherical harmonics, implemented in SCALE3 ABSPACK scaling algorithm.	*k* = -23→25
T_{min} = 0.837, T_{max} = 1.000	*l* = -18→15
10414 measured reflections	

Refinement

Refinement on F^2	Hydrogen site location: inferred from neighbouring sites
Least-squares matrix: full	H-atom parameters constrained
$R[F^2 > 2\sigma(F^2)]$ = 0.025	$w = 1/[\sigma^2(F_o^2) + (0.0356P)^2 + 7.2602P]$ where $P = (F_o^2 + 2F_c^2)/3$
$wR(F^2)$ = 0.065	$(\Delta/\sigma)_{max}$ < 0.001
S = 1.05	$\Delta\rangle_{max}$ = 0.18 e Å^{-3}
3201 reflections	$\Delta\rangle_{min}$ = -0.23 e Å^{-3}
230 parameters	Absolute structure: Flack x determined using 1163 quotients [(I+)-(I-)]/[(I+)+(I-)] (Parsons, Flack and Wagner, Acta Cryst. B69 (2013) 249-259).
1 restraint	Absolute structure parameter: -0.024 (2)

Special details

Geometry. All esds (except the esd in the dihedral angle between two l.s. planes) are estimated

using the full covariance matrix. The cell esds are taken into account individually in the estimation of esds in distances, angles and torsion angles; correlations between esds in cell parameters are only used when they are defined by crystal symmetry. An approximate (isotropic) treatment of cell esds is used for estimating esds involving l.s. planes.

Symmetry code: (i) -*x*+1, -*y*+1, *z*.

Document origin: *publCIF* [Westrip, S. P. (2010). *J. Apply. Cryst.*, **43**, 920-925].

Crystal data	
Chemical formula	$C_{56}H_{80}Co_2N_4$·1[C6H$_6$]·0.5[C6H$_{12}$]
M_r	1047.28
Crystal system, space group	Trigonal, $P\bar{3}$
Temperature (K)	100
a, *c* (Å)	28.1977 (8), 13.2243 (2)
V (Å^3)	9106.1 (5)
Z	6
Radiation type	Cu *K*α
μ (mm^{-1})	4.57
Crystal size (mm)	0.37 × 0.31 × 0.26
Data collection	
Diffractometer	XtaLAB Synergy, Dualflex, HyPix-Arc 150
Absorption correction	Multi-scan<br>*CrysAlis PRO* 1.171.43.143a (Rigaku Oxford Diffraction, 2024) Empirical absorption correction using spherical harmonics, implemented in SCALE3 ABSPACK scaling algorithm.
T_{min}, T_{max}	0.772, 1.000
No. of measured, independent and observed [$I > 2\sigma(I)$] reflections	60136, 12132, 7456
R_{int}	0.068
(sin θ/λ)$_{max}$ (Å^{-1})	0.630
Refinement	
$R[F^2 > 2\sigma(F^2)]$, $wR(F^2)$, S	0.070, 0.220, 1.05
No. of reflections	12132
No. of parameters	604

No. of restraints	48
H-atom treatment	H-atom parameters constrained
$\Delta\rangle_{max}$, $\Delta\rangle_{min}$ (e Å^{-3})	0.58, -0.53

Computer programs: *CrysAlis PRO* 1.171.43.143a (Rigaku OD, 2024), SHELXT 2018/2 (Sheldrick, 2018), *SHELXL* 2018/3 (Sheldrick, 2015), Olex2 1.5 (Dolomanov *et al.*, 2009).

References

Dolomanov, O. V., Bourhis, L. J., Gildea, R. J., Howard, J. A. K. & Puschmann, H. (2009). *J. Appl. Cryst.* **42**, 339–341.

Sheldrick, G. M. (2015). *Acta Cryst.* C**71**, 3–8.

Document origin: *publCIF* [Westrip, S. P. (2010). *J. Apply. Cryst.*, **43**, 920-925].

[(^{iPr}DI)Co(μ-H)$_2$]$_2$

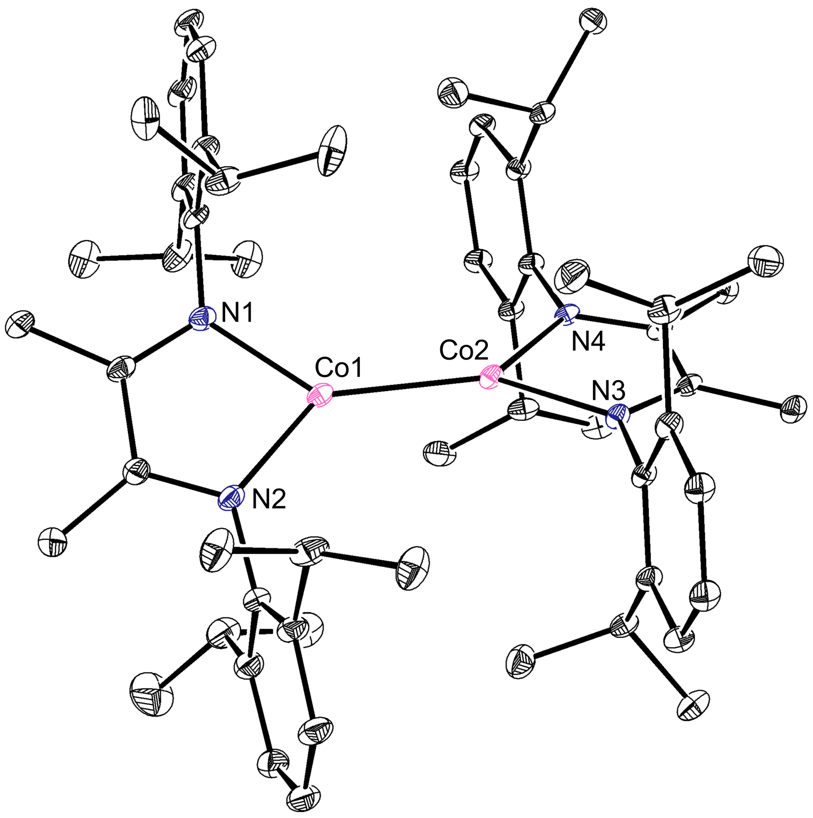

Computing details

Data collection: *CrysAlis PRO* 1.171.43.143a (Rigaku OD, 2024); cell refinement: *CrysAlis PRO* 1.171.43.143a (Rigaku OD, 2024); data reduction: *CrysAlis PRO* 1.171.43.143a (Rigaku OD, 2024); program(s) used to solve structure: SHELXT 2018/2 (Sheldrick, 2018); program(s) used to refine structure: *SHELXL* 2018/3 (Sheldrick, 2015); molecular graphics: Olex2 1.5 (Dolomanov *et al.*, 2009); software used to prepare material for publication: Olex2 1.5 (Dolomanov *et al.*, 2009).

(klm17_auto)

Crystal data

$C_{56}H_{80}Co_2N_4$·1[C6H$_6$]·0.5[C6H$_{12}$]	D_x = 1.146 Mg m^{-3}
M_r = 1047.28	Cu *K*α radiation, λ = 1.54184 Å
Trigonal, $P\bar{3}$	Cell parameters from 15025 reflections
a = 28.1977 (8) Å	θ = 3.1–75.1°
c = 13.2243 (2) Å	μ = 4.57 mm^{-1}

V = 9106.1 (5) Å^3	T = 100 K
Z = 6	Plate, dark brown
F(000) = 3384	0.37 × 0.31 × 0.26 mm

Data collection

XtaLAB Synergy, Dualflex, HyPix-Arc 150 diffractometer	12132 independent reflections
Radiation source: micro-focus sealed X-ray tube, PhotonJet (Cu) X-ray Source	7456 reflections with $I > 2\sigma(I)$
Mirror monochromator	R_{int} = 0.068
Detector resolution: 10.0000 pixels mm^{-1}	θ_{max} = 76.3°, θ_{min} = 3.1°
ω scans	h = -35→33
Absorption correction: multi-scan *CrysAlis PRO* 1.171.43.143a (Rigaku Oxford Diffraction, 2024) Empirical absorption correction using spherical harmonics, implemented in SCALE3 ABSPACK scaling algorithm.	k = -34→32
T_{min} = 0.772, T_{max} = 1.000	l = -16→15
60136 measured reflections	

Refinement

Refinement on F^2	48 restraints
Least-squares matrix: full	Hydrogen site location: inferred from neighbouring sites
$R[F^2 > 2\sigma(F^2)]$ = 0.070	H-atom parameters constrained
$wR(F^2)$ = 0.220	$w = 1/[\sigma^2(F_o^2) + (0.1305P)^2]$ where $P = (F_o^2 + 2F_c^2)/3$
S = 1.05	$(\Delta/\sigma)_{max}$ = 0.002
12132 reflections	$\Delta\rangle_{max}$ = 0.58 e Å^{-3}
604 parameters	$\Delta\rangle_{min}$ = -0.53 e Å^{-3}

Special details

Geometry. All esds (except the esd in the dihedral angle between two l.s. planes) are estimated using the full covariance matrix. The cell esds are taken into account individually in the estimation of esds in distances, angles and torsion angles; correlations between esds in cell parameters are only used when they are defined by crystal symmetry. An approximate (isotropic) treatment of cell esds is used for estimating esds involving l.s. planes.

Document origin: *publCIF* [Westrip, S. P. (2010). *J. Apply. Cryst.*, **43**, 920-925].

($(^{iPr}DI)Co(\eta^5\text{-}C_6H_6)$)

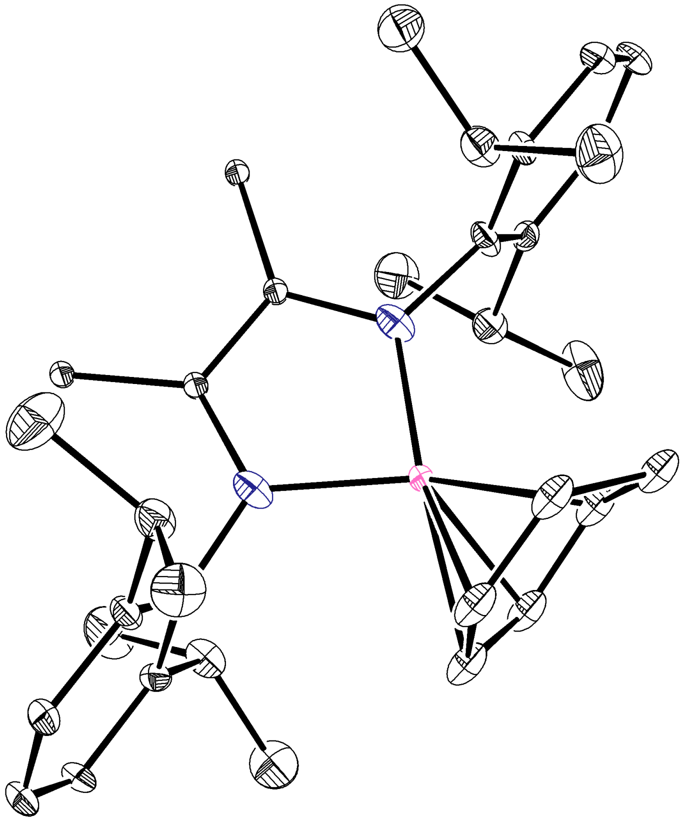

Computing details

Data collection: Bruker *APEX2*; cell refinement: Bruker *SAINT*; data reduction: Bruker *SAINT*; program(s) used to solve structure: *SIR* 92 (Giacavazzo, 1993); program(s) used to refine structure: *SHELXL2014* (Sheldrick, 2014); molecular graphics: *ORTEP* 3 (Farraguia, 1997).

(wnp53)

Crystal data

$C_{34}H_{44}CoN_2$	$Z = 1$
$M_r = 540.10$	$F(000) = 289.4$
Triclinic, $P1$	$D_x = 1.180$ Mg m^{-3}
$a = 9.0756$ (6) Å	Mo $K\alpha$ radiation, $\lambda = 0.71073$ Å
$b = 9.3818$ (6) Å	Cell parameters from 9592 reflections
$c = 10.3822$ (7) Å	$\theta = 2.5–32.0°$

α = 69.238 (3)°	μ = 0.59 mm^{-1}
β = 82.955 (4)°	T = 100 K
γ = 65.805 (4)°	Block, red
V = 753.71 (9) Å^3	0.25 × 0.22 × 0.19 mm

Data collection

Bruker APEX-II CCD diffractometer	9576 reflections with $I > 2\sigma(I)$
Radiation source: fine-focus sealed tube	R_{int} = 0.030
phi and ω scans	θ_{max} = 32.1°, θ_{min} = 2.5°
Absorption correction: multi-scan *SADABS* v2008/1 (Bruker AXS)	h = -13→13
T_{min} = 0.696, T_{max} = 0.746	k = -13→13
70843 measured reflections	l = -15→15
10412 independent reflections	

Refinement

Refinement on F^2	Hydrogen site location: inferred from neighbouring sites
Least-squares matrix: full	H atoms treated by a mixture of independent and constrained refinement
$R[F^2 > 2\sigma(F^2)]$ = 0.067	$w = 1/[\sigma^2(F_o^2) + (0.0622P)^2 + 1.0141P]$ where $P = (F_o^2 + 2F_c^2)/3$
$wR(F^2)$ = 0.169	$(\Delta/\sigma)_{max}$ < 0.001
S = 1.06	$\Delta\rangle_{max}$ = 1.19 e Å^{-3}
10412 reflections	$\Delta\rangle_{min}$ = -0.50 e Å^{-3}
343 parameters	Absolute structure: Refined as an inversion twin.
15 restraints	

Special details

Experimental. Collected, solved and refined by Iraklis Pappas.

Geometry. All esds (except the esd in the dihedral angle between two l.s. planes) are estimated using the full covariance matrix. The cell esds are taken into account individually in the estimation of esds in distances, angles and torsion angles; correlations between esds in cell parameters are only used when they are defined by crystal symmetry. An approximate (isotropic)

treatment of cell esds is used for estimating esds involving l.s. planes.

Refinement. Refined as a 2-component inversion twin.

(^{iPr}DI)Co(η^5-PhMe)

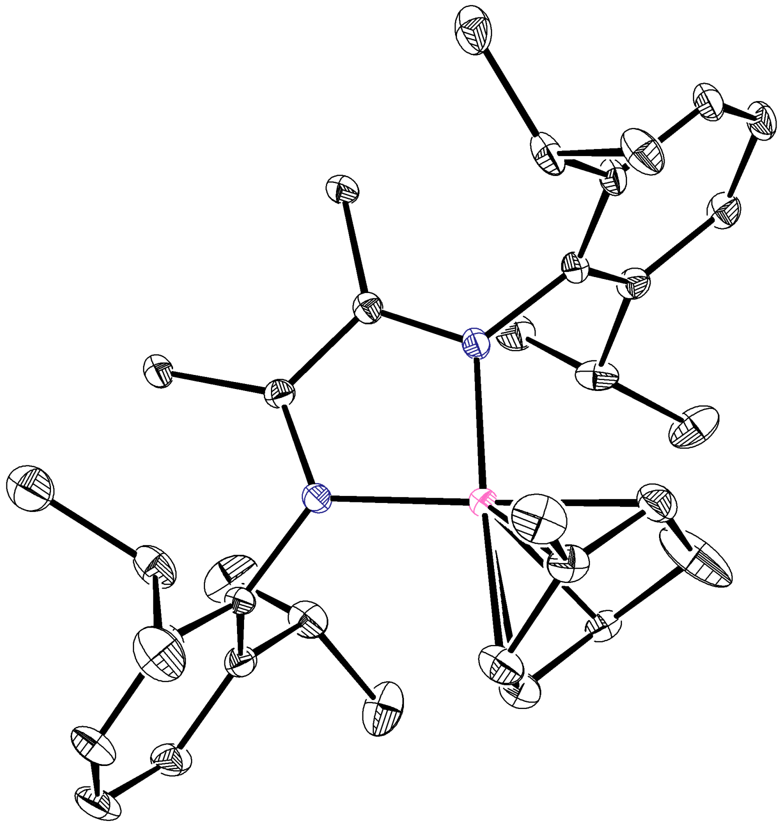

Computing details

Program(s) used to refine structure: *SHELXL2014* (Sheldrick, 2014).

(shelx)

Crystal data

$C_{17.50}H_{24.50}Co_{0.50}N$	$Z = 4$
$M_r = 278.34$	$F(000) = 600$
Triclinic, $P\bar{1}$	$D_x = 1.190$ Mg m^{-3}
$a = 10.1012$ (4) Å	Cu $K\alpha$ radiation, $\lambda = 1.54178$ Å
$b = 10.9893$ (4) Å	Cell parameters from 9960 reflections
$c = 14.3477$ (5) Å	$\theta = 3.1–66.7°$
$\alpha = 87.770$ (2)°	$\mu = 4.50$ mm^{-1}
$\beta = 88.213$ (2)°	$T = 100$ K

γ = 77.449 (2)°	Diamond, brown
V = 1553.01 (10) Å^3	0.38 × 0.24 × 0.22 mm

Data collection

Bruker D8 Venture Photon 100 CMOS diffractometer	5214 reflections with $I > 2\sigma(I)$
Radiation source: IµS microfocus source	R_{int} = 0.028
phi and ω scans	θ_{max} = 66.6°, θ_{min} = 3.1°
Absorption correction: multi-scan TWINABS BRUKER AXS	h = -12→12
T_{min} = 0.566, T_{max} = 0.753	k = -13→13
16220 measured reflections	l = -17→16
5370 independent reflections	

Refinement

Refinement on F^2	0 restraints
Least-squares matrix: full	Hydrogen site location: mixed
$R[F^2 > 2\sigma(F^2)]$ = 0.041	H atoms treated by a mixture of independent and constrained refinement
$wR(F^2)$ = 0.119	$w = 1/[\sigma^2(F_o^2) + (0.096P)^2 + 0.8689P]$ where $P = (F_o^2 + 2F_c^2)/3$
S = 0.91	$(\Delta/\sigma)_{max}$ < 0.001
5370 reflections	$\Delta\rangle_{max}$ = 0.52 e Å^{-3}
367 parameters	$\Delta\rangle_{min}$ = -0.56 e Å^{-3}

Special details

Experimental. Collected, solved and refined by Hongyu Aaron Zhong

Geometry. All esds (except the esd in the dihedral angle between two l.s. planes) are estimated using the full covariance matrix. The cell esds are taken into account individually in the estimation of esds in distances, angles and torsion angles; correlations between esds in cell parameters are only used when they are defined by crystal symmetry. An approximate (isotropic) treatment of cell esds is used for estimating esds involving l.s. planes.

(^{iPr}DI)Co(η^5-PhCF$_3$)

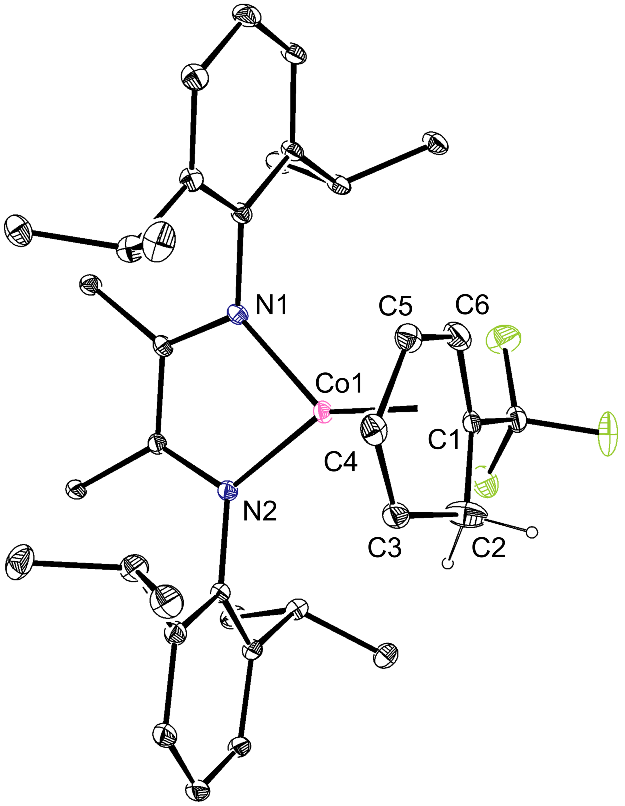

Computing details

Data collection: *CrysAlis PRO* 1.171.43.143a (Rigaku OD, 2024); cell refinement: *CrysAlis PRO* 1.171.43.143a (Rigaku OD, 2024); data reduction: *CrysAlis PRO* 1.171.43.143a (Rigaku OD, 2024); program(s) used to refine structure: *SHELXL* 2018/3 (Sheldrick, 2015); molecular graphics: Olex2 1.5 (Dolomanov *et al.*, 2009); software used to prepare material for publication: Olex2 1.5 (Dolomanov *et al.*, 2009).

(klm22_auto)

Crystal data

$C_{35}H_{46}CoF_3N_2$	$F(000) = 2592$
$M_r = 610.67$	$D_x = 1.274$ Mg m^{-3}
Monoclinic, $C2/c$	Cu $K\alpha$ radiation, $\lambda = 1.54184$ Å
$a = 34.5158$ (5) Å	Cell parameters from 18231 reflections
$b = 10.58744$ (15) Å	$\theta = 2.6–75.3°$

c = 17.6426 (3) Å	μ = 4.57 mm^{-1}
β = 98.8826 (16)°	T = 100 K
V = 6369.86 (17) $Å^3$	Plate, yellow
Z = 8	0.30 × 0.20 × 0.16 mm

Data collection

XtaLAB Synergy, Dualflex, HyPix-Arc 150 diffractometer	6477 independent reflections
Radiation source: micro-focus sealed X-ray tube, PhotonJet (Cu) X-ray Source	5208 reflections with $I > 2\sigma(I)$
Mirror monochromator	R_{int} = 0.132
Detector resolution: 10.0000 pixels mm^{-1}	θ_{max} = 75.8°, θ_{min} = 2.6°
ω scans	h = -42→34
Absorption correction: multi-scan *CrysAlis PRO* 1.171.43.143a (Rigaku Oxford Diffraction, 2024) Empirical absorption correction using spherical harmonics, implemented in SCALE3 ABSPACK scaling algorithm.	k = -13→13
T_{min} = 0.456, T_{max} = 1.000	l = -22→21
53681 measured reflections	

Refinement

Refinement on F^2	39 restraints
Least-squares matrix: full	Hydrogen site location: inferred from neighbouring sites
$R[F^2 > 2\sigma(F^2)]$ = 0.071	H-atom parameters constrained
$wR(F^2)$ = 0.209	$w = 1/[\sigma^2(F_o^2) + (0.1567P)^2]$ where $P = (F_o^2 + 2F_c^2)/3$
S = 1.05	$(\Delta/\sigma)_{max}$ = 0.001
6477 reflections	$\Delta\rangle_{max}$ = 1.38 e $Å^{-3}$
408 parameters	$\Delta\rangle_{min}$ = -0.76 e $Å^{-3}$

Special details

Geometry. All esds (except the esd in the dihedral angle between two l.s. planes) are estimated using the full covariance matrix. The cell esds are taken into account individually in the estimation of esds in distances, angles and torsion angles; correlations between esds in cell parameters are only used when they are defined by crystal symmetry. An approximate (isotropic) treatment of cell esds is used for estimating esds involving l.s. planes.

Document origin: *publCIF* [Westrip, S. P. (2010). *J. Apply. Cryst.*, **43**, 920-9

VII. References

1. Pangborn, A. B.; Giardello, M. A.; Grubbs, R. H.; Rosen, R. K.; Timmers, F. J. Safe and Convenient Procedure for Solvent Purification. *Organometallics* **1996**, *15*, 1518–1520.

2. (a) Bhadbhade, M.; Clentsmith, G.K.B.; Field, L.D. *Organometallics*, **2010**, 29, 6509-6517. (b) tom Dieck, H; Svoboda, M.; Greiser, T.Z. *Naturforsch., B: Chem. Sci.* **1981**, 36, 823-832.

3. Rosa, V.; Carabineiro, S.A.; Avilés, T.; Gomes, P.T.; Welter, R.; Campos, J.M.; Ribiero, M.R. Synthesis, characterisation and solid state structures of α-diimine cobalt(II) complexes: Ethylene polymerisation tests. *J. Organomet. Chem.*, **2008**, 693, 769-775.

4. Wang, X.; Fan, X.; Zhao, Y.; Wang, X.; Liu, B.; Su, J.; Dong, Q.; Xu, M.; Wu, B. Synthesis and Characterization of Cobalt Complexes with Radical Anionic a-Diimine Ligands. *Organometallics*, **2013**, 32, 6945-6949.

5. Palmer, W.N.; Diao, T.; Pappas, I.; Chirik, P.J.; High-Activity Cobalt Catalysts for Alkene Hydroboration with Electronically Responsive Terpyridine and a-Diimine Ligands. *ACS Catal.*, **2014**, 5, 622-626.

6. Lebowitz, M.J.; Mendelsohn, L.N.; Zhong, H.; Pecoraro M.V.; Shevlin, M.; Chirik, P.J. Mechanistic Insights into Regioselective Arene Insertion using Bis(Phosphine) Cobalt (I) Hydrides to form 1,3-Cyclohexadienes. *Manuscript submitted*.

7. Fulmer, G. R.; Miller, A. J. M.; Sherden, N. H.; Gottlieb, H. E.; Nudelman, A.; Stoltz, B. M.; Bercaw, J. E.; Goldberg, K. I. NMR Chemical Shifts of Trace Impurities: Common Laboratory Solvents, Organics, and Gases in Deuterated Solvents Relevant to the Organometallic Chemist. *Organometallics* **2010**, *29*, 2176–2179.
